# Basal Forebrain Cholinergic Neurons Have Specific Characteristics during the Perinatal Period

**DOI:** 10.1523/ENEURO.0538-23.2024

**Published:** 2024-05-24

**Authors:** Natalia Lozovaya, Anice Moumen, Constance Hammond

**Affiliations:** ^1^B&A Therapeutics, Marseille 13009, France; ^2^Neurochlore, Marseille 13009, France

**Keywords:** basal forebrain, cholinergic neurons, early-firing, late-firing, nucleus basalis Meynert, substantia innominata

## Abstract

Cholinergic neurons of the basal forebrain represent the main source of cholinergic innervation of large parts of the neocortex and are involved in adults in the modulation of attention, memory, and arousal. During the first postnatal days, they play a crucial role in the development of cortical neurons and cortical cytoarchitecture. However, their characteristics, during this period have not been studied. To understand how they can fulfill this role, we investigated the morphological and electrophysiological maturation of cholinergic neurons of the substantia innominata–nucleus basalis of Meynert (SI/NBM) complex in the perinatal period in mice. We show that cholinergic neurons, whether or not they express gamma-aminobutyric acid (GABA) as a cotransmitter, are already functional at Embryonic Day 18. Until the end of the first postnatal week, they constitute a single population of neurons with a well developed dendritic tree, a spontaneous activity including bursting periods, and a short-latency response to depolarizations (early-firing). They are excited by both their GABAergic and glutamatergic afferents. During the second postnatal week, a second, less excitable, neuronal population emerges, with a longer delay response to depolarizations (late-firing), together with the hyperpolarizing action of GABA_A_ receptor-mediated currents. This classification into early-firing (40%) and late-firing (60%) neurons is again independent of the coexpression of GABAergic markers. These results strongly suggest that during the first postnatal week, the specific properties of developing SI/NBM cholinergic neurons allow them to spontaneously release acetylcholine (ACh), or ACh and GABA, into the developing cortex.

## Significance Statement

During early postnatal days, basal forebrain cholinergic neurons from the substantia innominata–nucleus basalis of Meynert (SI/NBM) complex play a crucial role in cortical development. However, their morphological and electrophysiological characteristics have not been studied during this period. Here we show that perinatal SI/NBM cholinergic neurons, whether they still express or have coexpressed GABAergic markers, belong to a single, homogeneous population of spontaneously active, early-firing, bursting neurons, activated by both glutamatergic and GABAergic afferents until the GABA_A_ receptor-mediated current polarity shift from excitatory to inhibitory has occurred. This early high excitability suggests that they can release acetylcholine (ACh) or ACh and GABA into the developing cortical layers and fulfill their well described role in the early development of cortical neurons and networks.

## Introduction

Cholinergic neurons distributed within the basal forebrain (BF) nuclei, including the medial septum, the vertical and horizontal diagonal band of Broca, and the substantia innominata–nucleus basalis of Meynert (SI/NBM) complex, provide the main source of cholinergic innervation of the neocortex (Mesulam et al., 1983; Rye et al., 1984; Wu et al., 2014; Zaborszky et al., 2015; Boskovic et al., 2019). They are implicated in the maintenance of the sleep–wake cycle and in the processes underlying arousal and attention modulation (Brown et al., 2012; Monosov et al., 2015; Ballinger et al., 2016; Zant et al., 2016). Adult BF cholinergic neurons exhibit heterogeneity based on molecular and electrophysiological features. A large subpopulation coexpresses the necessary cell machinery for GABA release, that is, the GABA synthetic enzyme, glutamate decarboxylase GAD65(*Gad2*), and the vesicular GABA transporter (*Slc32 a1*). Acetylcholine (ACh) and GABA are cotransmitters since optogenetic stimulation of cholinergic fibers evokes monosynaptic synaptic currents mediated by both nicotinic ACh (nAChRs) and GABA_A_ receptors (GABA_A_Rs) in Layer 1 cortical interneurons. The GABA_A_R-mediated component disappears after selective conditional knock-out of *Slc32a1* in cholinergic neurons (Kosaka et al., 1988; Tkatch et al., 1998; Grill et al., 2004; Henny and Jones, 2008; Saunders et al., 2015; Granger et al., 2016; Case et al., 2017). Additionally, two distinct functional cholinergic cell types, the “early-firing” and “late-firing” neurons, are described in vitro and in vivo in adult mouse BF (Unal et al., 2012; Laszlovszky et al., 2020). In vivo, “early-firing” neurons are burst-firing neurons that can synchronize, whereas “late-firing” neurons are nonsynchronized regular firing neurons. They are likely to perform different roles in adult cortical activity and behavior (Laszlovszky et al., 2020). A possible correlation between the presence or absence of GABA as a cotransmitter and the two distinct functional populations has not been studied.

During early postnatal days, BF cholinergic neurons play a crucial role in cortical development. Growth cone-tipped choline acetyl transferase (ChAT)-positive axons are present in the subplate of frontal, parietal, and occipital cortices of rat pups from birth (P0) onward (Mechawar and Descarries, 2001). In the mouse pups, the density of acetylcholinesterase (AChE)-stained fibers increases significantly throughout the cortex from P0 to the second postnatal day (P2) and most of the AChE-positive fibers are located below the cortical plate, with some staining visible in the molecular layer (Höhmann and Ebner, 1985). Between P4 and P16, the number of varicosities per micrometer of ChAT-immunostained axon increases sharply in all cortical areas and layers and stabilizes thereafter to reach adult values (see review in Bruel-Jungerman et al., 2011). Moreover, functional muscarinic and calcium-permeable nAChRs are already present in the developing mouse cortex before birth (Atluri et al., 2001; Hohmann, 2003). Nonselective electrolytic or immunotoxic lesions of the NBM cholinergic region performed in rat pups at P0/P2 or specific lesion of NBM cholinergic neurons at P4 all result in delayed cortical neuronal development and permanently altered cortical cytoarchitecture and cognitive behaviors. This includes a reduced size of soma, dendritic abnormalities, altered connectivity, and synaptic plasticity (Hohmann et al., 1988; Hohmann and Berger-Sweeney, 1998; Robertson et al., 1998; Berger-Sweeney et al., 2001; Ricceri et al., 2002; Hohmann, 2003; Kuczewski et al., 2005). However, morphological and electrophysiological characteristics of BF cholinergic neurons have not been studied during this perinatal period.

To better understand why SI/NBM cholinergic neurons have such an impact on the development of cortical neurons and networks during the first postnatal days, we investigated their morphological and functional characteristics from Embryonic Day 18 (E18) to P15. We aimed at identifying the extent of their dendritic tree, their membrane properties, the impact of their afferent connections, and their type of ongoing activity. With the use of transgenic Lhx6-iCre^+/−^;RCE-EGFP^+/−^ mice, we could separately record and label in the SI/NBM region, the two cholinergic populations, and the cholinergic/GABAergic (ChAT^+^ EGFP^+^) and cholinergic/non-GABAergic (ChAT^+^ EGFP^−^) neurons (Lozovaya et al., 2018).

## Materials and Methods

### Animals

We generated transgenic mice Lhx6-iCre^+/−^;RCE-EGFP^+/−^ by crossing Lhx6-iCre^+/−^;RCE-EGFP^+/−^ mice (a generous gift from Prof. Gordon J. Fishell) with wild-type Swiss mice (CE Janvier). Vaginal plugs were checked early the following morning at 7 A.M. and noted as E0.5 of gestation. Mice were maintained on a 12 h light cycle (7 A.M.–7 P.M.) with *ad libitum* access to food and water. Experiments were performed in both males and females, in agreement with the European Community Council Directives (2010/63/UE). Protocols were approved by the local French ethical committee for animal experimentation (19196) and validated by the French Ministry of Higher Education, Research and Innovation (authorization 19196-2018071214167976).

We used slices from Lhx6-iCre^+/−^;RCE-EGFP^+/−^ mice, because EGFP expression allowed characterizing GABA/ACh neurons after post hoc ChAT immunocytochemistry. EGFP expression also allowed delineating the border of the globus pallidus (GP) since all GP cells are EGFP+ and fluorescent. This was particularly important in very young animals.

#### Slice preparation and patch-clamp recordings

For prenatal date E18, we killed pregnant dams by cervical dislocation and rapidly (in 20–30 s) isolated uterine horns from their blood supply. We immediately delivered and decapitated pups. We removed fetuses from timed-pregnant females 24 ± 6 h before the estimated day of delivery. For postnatal dates P0–P5, we quickly killed pups by decapitation, but after P5 we anesthetized pups with isoflurane before decapitation. We kept brains in ice-cold oxygenated solution containing the following (in mM), 118 choline chloride, 2.5 KCl, 0.7 CaCl_2_, 7 MgCl_2_, 1.2 NaH_2_PO_4_, 26 NaHCO_3_, and 8 glucose, and performed parasagittal slices (300–350 μm thick) using a vibratome (VT1200 Leica Microsystems). During the recovery period (at least 2 h), slices were placed at room temperature (RT, 22–25°C) in standard artificial cerebrospinal fluid (ACSF) saturated with 95% O_2_/5% CO_2_ and containing the following (in mM): 125 NaCl, 3.5 KCl, 0.5 CaCl_2_, 3 MgCl_2_, 1.25 NaH_2_PO_4_, 26 NaHCO_3,_ and 10 glucose, 300 mOsml^−1^. For recordings, we used an ACSF of the same composition but containing 2 mM CaCl_2_ and 1 mM MgCl_2_. Slices were transferred to the recording chamber and perfused with oxygenated ACSF (3 ml/min) at RT. Neurons were visualized using infrared-differential interference optics (Microscope Olympus BX51WI). Patch pipettes were pulled from borosilicate glass capillaries (World Precision Instruments). In parasagittal slices from Lhx6-iCre;RCE-EGFP mice, we identified the dual cholinergic/GABAergic neurons as EGFP^+^ neurons and the cholinergic neurons as EGFP^−^ neurons with a large soma (≥40 µm diameter), thick primary dendrites, and a typical response to hyperpolarizing pulses. Their cholinergic nature was later confirmed with post hoc immunocytochemistry (see below, Immunohistochemistry, reconstruction of biocytin-filled neurons, and Sholl analysis). We performed cell-attached recordings 5–7 min after establishment of the gigaohm seal and after stabilization of the baseline with patch pipettes filled with extracellular solution. For whole-cell recordings, patch pipettes (World Precision Instruments) were filled with intracellular solution as follows (in mM): 130 K-gluconate, 10 Na-gluconate, 7 NaCl, 4 MgATP, 4 Na_2_-phosphocreatine, 10 HEPES, and 0.3 GTP, pH 7.3 (with KOH, 280 mOsml^−1^), and biocytin (final concentration of 0.3–0.5%). In whole-cell recordings, equilibrium potential for chloride ions was around −75 mV and equilibrium potential for AMPA channels ∼0–10 mV. We discarded cells with leakage current over 40–50 pA. Whole-cell recordings were performed with the EPC-10 amplifier and PATCHMASTER software v2 × 73.2 (HEKA Elektronik) and filtered at 3–10 kHz. To determine current–voltage (I–V) relationships and calculate input resistance of cells, we recorded voltage responses (current-clamp mode) to a series of 1 s current pulses. For the hyperpolarization-activated sag parameters, we measured the ratio between the peak amplitude and the amplitude at the end of the voltage response. We measured the half-width duration of action potentials (APs) at half the amplitude between the AP peak and threshold potentials. We measured the after-hyperpolarization (AHP) amplitude between AP threshold potentials and AHP peak. The Clampfit 10.6.2.2 (Molecular Devices), Igor 6.3.7.2 (WaveMetrics), and OriginPro software were used for analysis. Spontaneous postsynaptic outward currents were recorded at *V*_H_ = 0 to +10 mV closest to the reversal potential of AMPA receptor-mediated currents, and spontaneous postsynaptic inward currents were recorded at −70 mV, close to the reversal equilibrium potential of chloride ions.

#### Immunohistochemistry, reconstruction of biocytin-filled neurons, and Sholl analysis

Parasagittal slices were incubated with ChAT primary antibody coupled with a donkey anti-goat Alexa Fluor 633 (1:500, The Jackson Laboratories). After 20–24 h fixation in 4% paraformaldehyde (Alfa Aesar, Thermo Fisher Scientific) in phosphate-buffered saline (PBS; Invitrogen, Thermo Fisher Scientific) at 4°C, slices were washed three times in PBS, and after a 1 h preincubation in PBS containing 10% normal donkey serum (NDS; Jackson ImmunoResearch Laboratories), 1% bovine serum albumin (BSA; Sigma-Aldrich, Merck KGaA), and 0.3% Triton X-100 (Sigma-Aldrich, Merck KGaA), they were incubated overnight at 4°C with goat anti-ChAT antibody (AB144P, Chemicon, Merck KGaA; 1:300) in PBS containing 1% NDS, 1% BSA, and 0.3% Triton X-100. Twenty-four hours after, slices were washed three times in PBS and incubated in PBS containing 1% BSA with a donkey anti-goat coupled to Alexa Fluor 555 (A-21432, Invitrogen, Thermo Fisher Scientific; 1:500) and streptavidin coupled to Alexa Fluor 647 (S32357, Invitrogen, Thermo Fisher Scientific; 1:500). Slices were then washed three times in PBS and coverslipped using a Fluoromount-G mounting medium (Invitrogen, Thermo Fisher Scientific). We followed a similar procedure to perform Lhx6 staining. We used a mouse anti-Lhx6 (1:50,000, Santa Cruz Biotechnology, ref. sc-271433) and a donkey anti-mouse antibody coupled to Alexa Fluor 647 (1:500, Invitrogen). For biocytin-filled cell localization, identification, and reconstruction, confocal images were acquired on a SP8X Leica microscope (Leica Microsystems) using a diode 405 for the excitation of Hoechst or Alexa Fluor 405 (5%, spectral detection 415–460 nm), an OPSL 488 for the excitation of EGFP or Alexa Fluor 488 (10%, spectral detection 498–528 nm), an OPSL 552 for the excitation of Alexa Fluor 555 (5%, spectral detection 559–600 nm), and a diode 638 for the excitation of Alexa Fluor 647 (0.1%, spectral detection 649–776 nm). Images were acquired at 200 Hz using a 10× objective (for cells localization; pixel size, 2.2705 µm) and a 40× oil immersion objective (for cell identification and reconstruction; pixel size, 0.2838135 µm), pinhole set to “Airy 1,” by scanning with a *z* step of 0.5016 μm when needed. Images were edited using Photoshop and Illustrator (Adobe Systems Software Ireland). After image acquisition, stacks were imported in the open-source platform Fiji (https://fiji.sc/) and processed for neuron reconstruction and Sholl analysis (Lozovaya et al., 2018).

**Figure 1.**
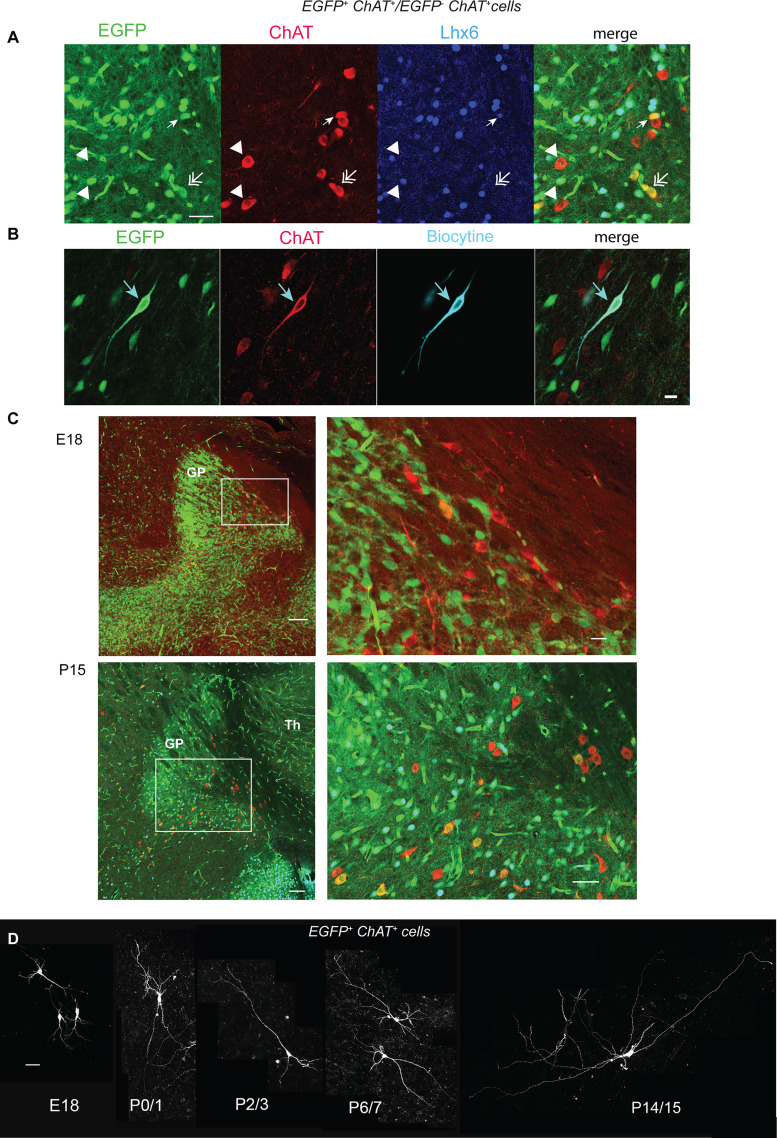
A subset of NBM cholinergic neurons expresses cholinergic (ChAT) and GABAergic (EGFP, Lhx6) markers in sagittal slices from Lhx6-iCre;RCE-EGFP mice. ***A***, ChAT (red) and Lhx6 (blue) coimmunolabeling of a NBM neuron expressing EGFP (EGFP^+^, green, arrow). The double arrow points to an EFGP^+^ ChAT^+^ Lhx6^−^ neuron, and the two arrow heads point down to two EFGP^−^ ChAT^+^ Lhx6^−^ neurons of the same region. ***B***, Example of EGFP expression (green) and ChAT staining (red) in a biocytin-filled (blue) neuron (arrow). ***C***, EGFP expression (green) and ChAT staining (red) in a subset of NBM neurons at E18 and P15. On the right are the enlargements of the NBM region as indicated on the left. ***D***, Representative biocytin-filled cholinergic/GABAergic (EFGP^+^ ChAT^+^) neurons at the indicated ages. Scale bars: ***A***, 50 µm; ***B***, 50 µm; ***C***, E18, 100 µm, 20 µm; P15, 200 µm, 100 µm; ***D***, 50 µm.

To confirm the cholinergic identity of the recorded neurons, we revealed the biocytin injected during whole-cell recordings and performed post hoc ChAT immunohistochemistry. Recorded (biocytin-positive) neurons that were EGFP positive (EGFP^+^) and ChAT positive (ChAT^+^) were cholinergic neurons that have expressed (Lhx6^−^) or still express (Lhx6^+^) the LIM homeodomain 6 (Lhx6; Fig. 1*A–C*). Biocytin-positive neurons that were EGFP negative (EGFP^−^) and ChAT^+^ were cholinergic neurons that have never expressed Lhx6 (Lhx6^−^; Fig. 1*A–C*). The starting date of our study was E18 since it was the earliest date for optimal reliability of ChAT immunostaining (Fig. 1*C*).

### Drugs

2,3-Dioxo-6-nitro-1,2,3,4-tetrahydrobenzo[*f*]quinoxaline-7-sulfonamide (10 µM, Tocris Bioscience, Ref. 0373), isoguvacine (10 μM, Sigma-Aldrich, Ref. G002), DL-2-Amino-5-phosphonovaleric acid (40 µM, Sigma-Aldrich, Ref. A5282), picrotoxin (50 µM, Tocris Bioscience, Ref. 1128), bumetanide (10 μM, Sigma-Aldrich, Ref. B3023).

### Statistical analysis and cluster analysis

Data from morphological and electrophysiological experiments were computed in Prism 8 (GraphPad Software) or OriginPro 019 (64-bit) 9.6.0.172 (OriginLab). They were tested for normality and homoscedasticity using the Shapiro–Wilk and Leven's tests before performing statistical analysis. We analyzed morphological and electrophysiological data using a two-tailed *t* test or Mann–Whitney test. For multiple comparisons we used one-way ANOVA test followed by Fisher's LSD post hoc test or one-way ANOVA/Kruskal–Wallis test followed by Dunn's multiple-comparison post hoc test. We calculated means by averaging data from *n* cells. We also indicated the number of animals *N* for each dataset in the figure legends. We performed confidence interval estimation (95%) and effect size using Estimation Stats (https://www.estimationstats.com/; Claridge-Chang and Assam, 2016; Ho et al., 2019) and statistical power analysis using OriginPro (OriginLab) and Anastats (Anastats, www.anastats.fr). All data are presented as means ± SEM (*p < 0.05; **p < 0.01; ***p < 0.001). We rejected null hypothesis when *P* was inferior to 0.05 and 95% CI did not contain zero. All data are included in Extended Data Tables 2-1, 3-1, 4-1, 5-1, 6-1, and 7-1. Data availability statement: all relevant data are available from the corresponding authors upon reasonable request.

For cluster analysis, we performed hierarchical clustering, *k*-means and principal component analysis (PCA) with Origin (OriginLab). We first performed hierarchical clustering using Euclidian distance metrics and group average searching to group neuronal populations. The electrophysiological parameters tested for this analysis were input resistance, delay of the first spike in response to juxtathreshold depolarization, amplitude and half-width of evoked AP, AP maximal frequency, *V*_Thresh_, AHP amplitude, and sag amplitude in response to −50 and −100 pA. We then performed a PCA to identify the correlation between the different analyzed features and applied the screen plot “elbow” criterion to determine the number of clusters. To detect and characterize the subpopulation of EGFP^+^ and EGFP^−^ cholinergic cells, we performed *k*-means clustering based on selected parameters. *K*-Means silhouette indexes, specialized for measurement cluster quality (goodness), were calculated with Orange Data Mining Toolbox in Python (Demsar et al., 2014) to assess the cluster quality and validate the consistency within the cluster of data. The statistics of each cluster were used to characterize subpopulations and determine their phenotype, later named Clusters 1 and 2. We then compared results obtained with the two methods.

## Results

Using Lhx6-iCre;RCE-EGFP mice (E18-P15) and post hoc ChAT immunohistochemistry, we identified ChAT^+^ EGFP^+^ and ChAT^+^ EGFP^−^ neurons in the SI/NBM region (Fig. 1*A–C*; Extended Data Fig. 2-2*A*). We recorded 182 ChAT^+^ EGFP^+^ and 78 ChAT^+^ EGFP^−^ cholinergic neurons in the SI/NBM region from 79 Lhx6-iCre;RCE-EGFP mice. Throughout the text, cholinergic/GABAergic neurons are ChAT^+^ EGFP^+^ neurons previously shown to coexpress GABAergic markers (Lozovaya et al., 2018), whereas cholinergic/non-GABAergic neurons are ChAT^+^ EGFP^−^ neurons. We mainly focused the present study on ChAT^+^ EGFP^+^ neurons and compared some of their characteristics to ChAT^+^ EGFP^−^ neurons.

### Cholinergic/GABAergic neurons constitute a single population which rapidly develop during the first postnatal week

**Figure 2.**
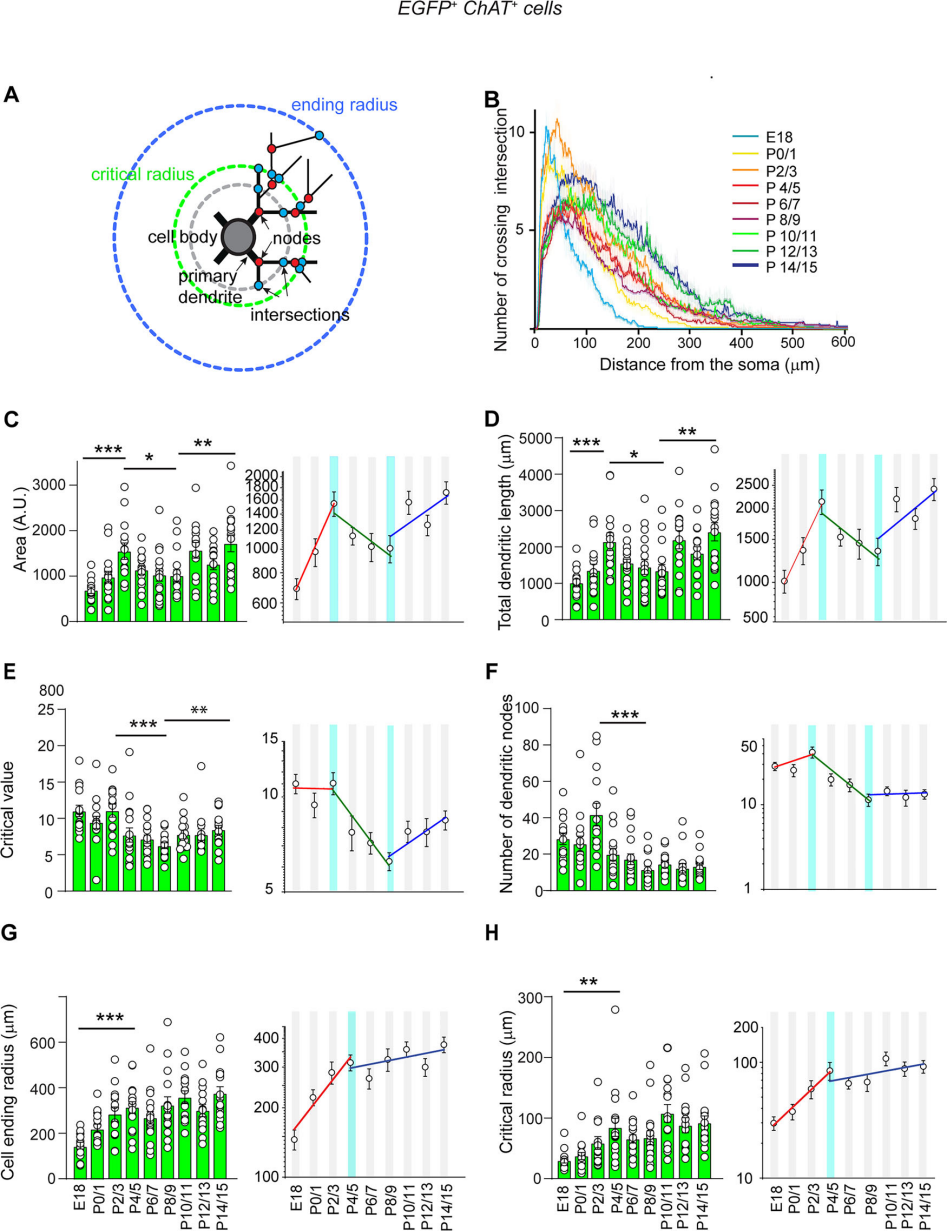
Development of the morphological properties of cholinergic/GABAergic neurons (E18–P15). ***A***, Schematic representation of the quantified morphological parameters; the Sholl profiles (***B***) and morphological parameters (***C–H***) were obtained by overlaying a template of concentric and equidistant circles onto a two-dimensional images of biocytin-filled neurons with the first circle centered on the soma (radius 0): nodes (red dots), the points of branching of dendrites; crossing intersections (blue dots), the points where the dendritic branches intersect the Sholl circles; critical radius (green), the radius of the circle with the maximum number of crossing intersections; critical value, the number of crossing intersections at critical radius; ending radius (blue), the maximal radius of the circle with crossing intersection(s); the Sholl profile is the number of crossing intersections as a function of the radial distance from the cell soma. ***B***, Mean Sholl profiles at the indicated ages. ***C–H***, Left, Quantification of the main morphological properties (as indicated in ordinates) during development (abscissae). ***C–H***, Right, Linear fits of fast (red) changes of the same morphological parameters in semilog *Y* coordinates. The light blue vertical bar indicates the peaks of development. E18: *n* = 15, *N* = 4; P0/1: *n* = 15, *N* = 7; P2/3: *n* = 14, *N* = 6; P4/5: *n* = 17, *N* = 6; P6/7: *n* = 17, *N* = 6; P8/9: *n* = 16, *N* = 5; P10/11: *n* = 14, *N* = 6; P12/13: *n* = 14, *N* = 6; P14/15: *n* = 16, *N* = 7. *n*, number of cells; *N*, number of mice. Mean ± SEM, *p < 0.05; **p < 0.01; ***p < 0.001 (Extended Data Table 2-1). Comparison of the dendritic parameters of GABAergic and non-GABAergic cholinergic neurons is presented in Extended Data Figure 2-2.

Figure 1*D* illustrates the typical perinatal development of dendritic trees of cholinergic/GABAergic neurons. Sholl analysis of the density profiles of dendritic branches of 3D-reconstructed, biocytin-filled, cholinergic/GABAergic neurons, as a function of distance from the soma (Fig. 2*A*,*B*), showed the initial exponential increase of all parameters (except critical value, i.e., the maximal number of dendritic intersections) from E18 to P2/3 or P4/5. Then, the area under the curve, total dendritic length, critical value, and number of dendritic nodes exponentially decreased from P2/3 to P8/9 before increasing again or stabilizing (Fig. 2*B*–*H*; Extended Data Table 2-1). These synchronous decreases probably reflected dendritic pruning (see Discussion). We did not detect significant change in the number of primary dendrites from E18 (4.7 ± 0.7; *n* = 15) to P14/15 [3.6 ± 0.3 (*n* = 16), *p* = 0.17].

10.1523/ENEURO.0538-23.2024.t2-1 Table 2-1 **Statistical analysis related to Figure 2 and Extended Data Figure 2-2.** Summary of statistical tests for Figure 2 ***C-H*** and Extended Data Figure 2-2 ***E-K.*** 95% C.I. of diff - confidence interval for effect size. Download Table 2-1, DOCX file.

10.1523/ENEURO.0538-23.2024.f2-2 Figure 2-2 **Comparison of the dendritic parameters of GABAergic and non-GABAergic cholinergic neurons (P0-15). *A,*** Example of ChAT staining (red) in two EGFP^-^, biocytin-filled (blue) neurons (arrows) in a slice from a Lhx6-iCre;RCE^-^EGFP^+^ mouse. Scale bar: 30 µm***. B,*** Representative biocytin-filled cholinergic/GABAergic (arrows) and cholinergic/non-GABAergic (double arrow) neurons at P5. Scale bar: 50 µm. ***C,*** Mean Sholl profiles for cholinergic/GABAergic neurons, and ***D***, Mean Sholl profiles of cholinergic/non-GABAergic neurons at the indicated ages. ***E-K,*** Comparison of dendritic parameters (as indicated in ordinates and see Figure 2*A*) of biocytin-filled GABAergic (green) and non-GABAergic (grey) cholinergic neurons during development (abscissae). Cholinergic/GABAergic: (P0/1: n=15, N=7; P4/5: n= 17, N=6; P10/11: n=14, N=6; P14/15: n=16, N=7). Cholinergic/non-GABAergic:(P0/1: n=15, N=4; P4/5: n= 17, N=3; P10/11: n=13, N=3; P12/13: n=14, N=6; P14/15: n=12, N=6). n=number of cells, N=number of mice. Mean ± SEM, *: P < 0.05; **: P < 0.01; ***P: < 0.001. (Extended Data Table 2-1). Download Figure 2-2, TIF file.

**Figure 3.**
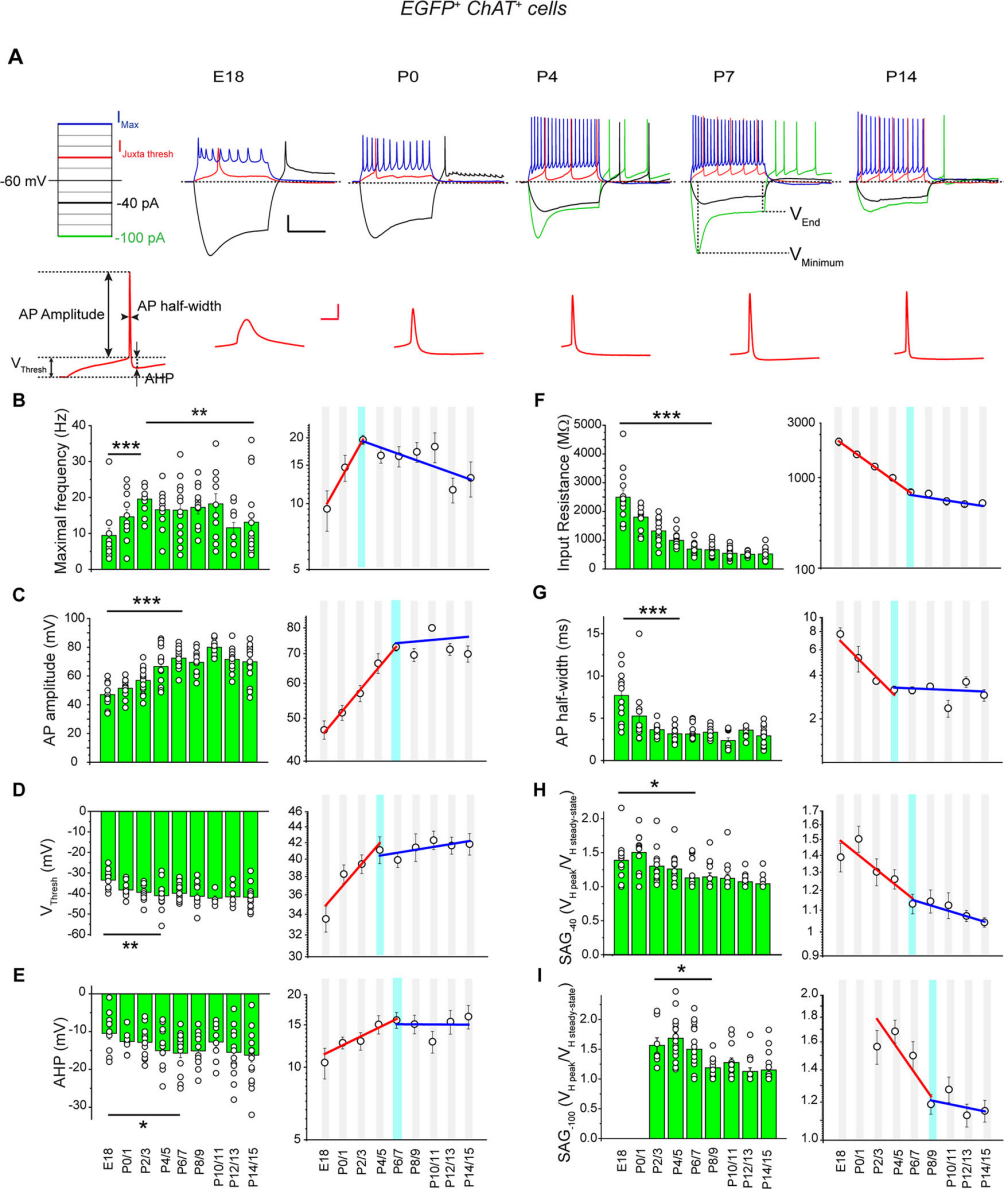
Development of the electrophysiological properties of cholinergic/GABAergic neurons (E18–P15). ***A***, Top, Representative voltage responses to a series of current pulses (whole-cell configuration). Current injection protocol (left) depicts the pulses used to obtain the traces on the right. Sag amplitude was measured in response to hyperpolarizing pulses of −40 pA (black, SAG_-40_) or of −100 pA (green, SAG_-100_) as the ratio of amplitude between *V*_minimum_ and *V*_end_; spike responses are displayed in response to juxtathreshold depolarizing (red) and maximal depolarizing (blue) current pulses. Bottom, Action potentials recorded in response to juxtathreshold depolarizing pulses at extended timescale (right) and the AP parameters measured (left). ***B–I***, Left columns, Quantification of the main intrinsic membrane properties (as indicated in ordinates) during development (abscissae). ***B–I*** *Right*, Linear fits of fast (red) changes of the electrophysiological parameters in semilog *Y* coordinates. The light blue vertical bar indicates the peak of fast development. ***B***, Mean maximal frequency. ***C***, Mean AP amplitude. ***D***, Mean AP threshold (*V*_Thresh_). ***E***, Mean AHP amplitude. ***F***, Mean input resistance. ***G***, Mean half-width of evoked APs. (E18: *n* = 13, *N* = 4; P0/1: *n* = 11, *N* = 4; P2/3: *n* = 14, *N* = 6; P4/5: *n* = 15, *N* = 7; P6/7: *n* = 19, *N* = 7; P8/9: *n* = 12, *N* = 4; P10/11: *n* = 13, *N* = 4; P12/13: *n* = 14, *N* = 3; P14/15: *n* = 17, *N* = 6). ***H***, Mean sag amplitude in response to a −40 pA hyperpolarizing pulse (Sag_-40_; E18: *n* = 12, *N* = 4; P0/1: *n* = 11, *N* = 4; P2/3: *n* = 12, *N* = 6; P4/5: *n* = 16, *N* = 8; P6/7: *n* = 18, *N* = 7; P8/9: *n* = 13, *N* = 5; P10/11: *n* = 13, *N* = 4; P12/13: *n* = 14, *N* = 3; P14/15: *n* = 17, *N* = 6). ***I***, Mean sag amplitude in response to a −100 pA hyperpolarizing pulse (Sag_-100_; P2/3: *n* = 8, *N* = 6; P4/5: *n* = 16, *N* = 8; P6/7: *n* = 15, *N* = 7; P8/9: *n* = 13, *N* = 5; P10/11: *n* = 13, *N* = 4; P12/13: *n* = 13, *N* = 3; P14/15: *n* = 17, *N* = 6). *n*, number of cells; *N*, number of mice. Mean ± SEM, *p < 0.05; **p < 0.01; ***p < 0.001 (Extended Data Table 3-1). Comparison of the intrinsic electrophysiological properties of GABAergic and non-GABAergic cholinergic neurons is presented in Extended Data Figure 3-2.

In response to depolarizing steps, cholinergic/GABAergic neurons generated spikes at E18 (*n* = 13 out of 13) with a mean maximal frequency of 9.5 ± 1.9 Hz (*n* = 13; Fig. 3*A*,*B*). Maximal spike frequency and spike parameters (threshold potential, peak amplitude, half-width, after spike hyperpolarization maximal amplitude), together with membrane resistance and the amplitude of the depolarizing “sag” displayed back toward the baseline during a hyperpolarizing current pulse, all exponentially increased or decreased to peak values during the first postnatal days (from E18 to P3/P7; Fig. 3*B*–*I*; Extended Data Table 3-1).

10.1523/ENEURO.0538-23.2024.t3-1 Table 3-1 **Statistical analysis related to Figure 3 and Extended Data Figure 3-2.** Summary of statistical tests for Figure 3 ***B-I*** and Extended Data Figure 3-2 ***A-H.*** 95% C.I. of diff - confidence interval for effect size. Download Table 3-1, DOCX file.

10.1523/ENEURO.0538-23.2024.f3-2 Figure 3-2 **Comparison of the intrinsic electrophysiological properties of GABAergic and non-GABAergic cholinergic neurons (P0-P15). *A-F***, Quantification of the main electrophysiological properties (as indicated in ordinates and see Figure 3*A*) of GABAergic (green) and non-GABAergic (grey) cholinergic neurons during development. (EGFP^+^: P0/1: n=11, N=4; P4/5: n= 15, N=7; P6/7: n= 19, N=7; P10/11: n=13, N=4; P14/15: n=17, N=6; EGFP^-^: P0/1: n=17, N=5; P4/5: n= 14, N=3; P10/11: n=13, N=3; P14/15: n=18, N=5).  ***G***, Mean sag amplitude in response to a -40 pA hyperpolarizing pulse (Sag_-40_), (EGFP^+^: P0/1: n=11, N=4; P4/5: n= 16, N=8; P10/11: n=13, N=4; P14/15: n=17, N=6; EGFP^-^: P0/1: n=17, N=5; P4/5: n= 13, N=3; P10/11: n=13, N=3; P14/15: n=18, N=5). ***H,*** Mean sag amplitude in response to a -100 pA hyperpolarizing pulse (Sag_-100_), (EGFP^+^: P4/5: n= 16, N=8; P10/11: n=13, N=4; P14/15: n=17, N=6; EGFP^-^: P4/5: n= 13, N=3; P10/11: n=13, N=3; P14/15: n=18, N=5) at the indicated ages. n=number of cells, N=number of mice. Mean ± SEM, *: P < 0.05; **: P < 0.01; ***P: < 0.001 (Extended Data Table 3-1). Download Figure 3-2, TIF file.

**Figure 4.**
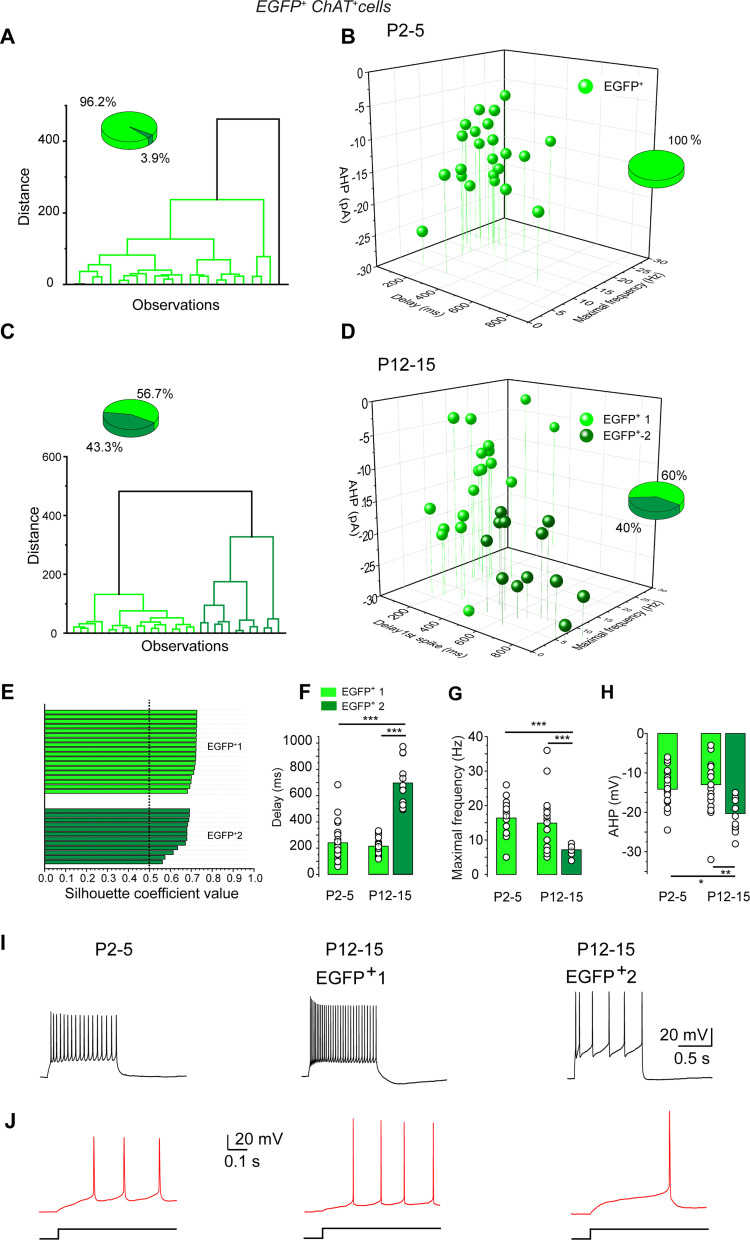
Cluster analysis of cholinergic/GABAergic neurons based on intrinsic electrophysiological properties at P2–5 and P12–15. ***A***, Hierarchical clustering dendrogram for intrinsic electrophysiological properties of P2–5 neurons (delay of the first spike in response to a juxtathreshold depolarization, AP amplitude, maximal frequency, *V*_Thresh_, AHP amplitude, half-width of evoked AP, Sag amplitudes in response to −40 and −100 pA). ***B***, 3D plot of the unique cluster of cholinergic/GABAergic (EGFP^+^, light green) neurons obtained from the *k*-means algorithm. Parameters: AHP amplitude, delay of the first spike in response to juxtathreshold depolarization and maximal frequency (P2–5; *n* = 23; *N* = 11). ***C***, Hierarchical clustering dendrogram for intrinsic electrophysiological properties (the same set of parameters as in ***A***) of P12–15 neurons (*n* = 30; *N* = 15). ***D***, 3D plot of the two clusters EGFP^+^1 (light green; *n* = 18; *N* = 9) and EGFP^+^2 (dark green; *n* = 12; *N* = 6) obtained from the *k*-means algorithm (P12–15). Parameters, AHP amplitude, delay of the first spike in response to juxtathreshold depolarization, and maximal frequency. Insets in ***A–D*** show percentage of cells belonging to each cluster. ***E***, Silhouette coefficient plot for EGFP^+^1 and EGFP^+^2. ***F–H***, Quantification of delay of the first spike in response to juxtathreshold depolarization (***F***), maximal frequency (***G***), and AHP amplitude (***H***), for datasets at P2–5 and at P12–15. *n*, number of cells, *N*, number of mice. Mean ± SEM; *p < 0.05; **p < 0.01; ***p < 0.001 (Extended Data Table 4-1). ***I***, Representative responses to maximal depolarizing current pulses (black). ***J***, Representative responses to the minimum current (rheobase) required to evoke an AP (red) at expanded time scale, for a P2–5 neuron and for two different P12–15 neurons belonging to the EGFP^+^1 or EGFP^+^2 cluster, as indicated. Cluster analysis of GABAergic and non-GABAergic cholinergic neurons (P2–5 and P12–15) based on intrinsic electrophysiological properties is presented in Extended Data Figure 4-2.

We next wanted to test whether cholinergic/GABAergic neurons constituted two functional populations during the early postnatal period as described in the adult (Unal et al., 2012; Laszlovszky et al., 2020). We performed hierarchical and *k*-means cluster analyses based on the electrophysiological parameters (see Materials and Methods). This revealed that 96% of P2/5 cholinergic/GABAergic neurons belonged to a single population of neurons mostly characterized by a short delay of the first spike to juxtathreshold depolarization, the “early-firing” neurons (Fig. 4*A*,*B*,*F*–*J*; Extended Data Table 4-1), in contrast to the P12/15 neuronal population (see below). Therefore, SI/NBM cholinergic/GABAergic neurons constitute a single population of early-firing neurons at P2–5.

10.1523/ENEURO.0538-23.2024.t4-1 Table 4-1 **Statistical analysis related to Figure 4 and Extended Data Figure 4-2.** Summary of statistical tests for Figure 4 ***F-H*** and Extended Data Figure 4-2 ***F-H.*** LCL-Lower Confidence Interval, UCL- Upper Confidence Interval Download Table 4-1, DOCX file.

**Figure 5.**
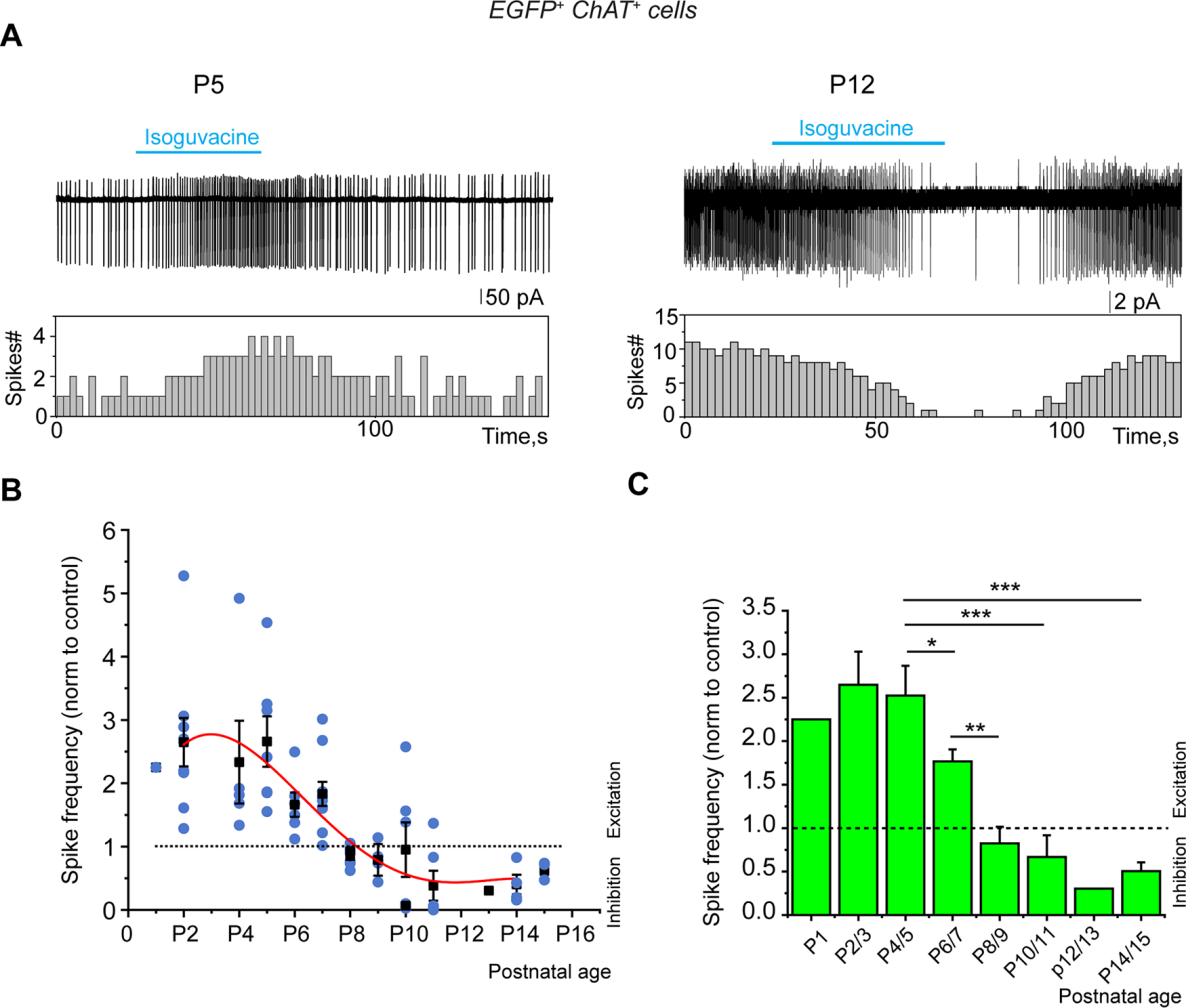
Developmental shift of the GABA_A_R-mediated response polarity, from excitatory to inhibitory, recorded from cholinergic/GABAergic neurons (P2–15). ***A***, Representative cell-attached recordings (top) and corresponding frequency histograms (bottom) of ongoing spike activity before, during, and after isoguvacine (10 μM) application at the indicated ages. ***B,C***, Mean values of isoguvacine maximal effects on spike frequency [P2/3: *n* = 9 (*N* = 3); P4/5: *n* = 12 (*N* = 5); P6/7: *n* = 16 (*N* = 4); P8/9: *n* = 12 (*N* = 4); P10/11: *n* = 12 (*N* = 3); P14/15: *n* = 5 (*N* = 3)]. *n*, number of cells; *N*, number of mice. Mean ± SEM; *p < 0.05; **p < 0.01; ***p < 0.001 (Extended Data Table 5-1).

10.1523/ENEURO.0538-23.2024.f4-2 Figure 4-2 **Cluster analysis** **of GABAergic and non-GABAergic cholinergic neurons (P2-5 and P12-15) based on intrinsic electrophysiological properties. *A***, Hierarchical clustering dendrogram for the intrinsic electrophysiological properties of P2-5 cholinergic/non-GABAergic neurons: delay of the 1st spike in response to a juxta threshold depolarization, AP amplitude, maximal frequency, V_Thresh_, AHP amplitude, half-width of evoked AP, Sag amplitudes in response to -40 pA and -100 pA. ***B,*** 3-D plot of the unique cluster of cholinergic/non-GABAergic neurons (EGFP^-^, grey) obtained from the k-means algorithm; Parameters: AHP amplitude, delay of the 1^st^ spike in response to juxta threshold depolarization and maximal frequency (P2-P5, n=14, N=3). The unique cluster of cholinergic/GABAergic neurons (EGFP^+^, green, see Fig. 5B) is superimposed. ***C,*** Hierarchical clustering dendrogram for the intrinsic electrophysiological properties of P12-15 cholinergic/non-GABAergic neurons: delay of the 1st spike in response to a juxta threshold depolarization, AP amplitude, maximal frequency, V_Thresh_, AHP amplitude, half-width of evoked AP, Sag amplitudes in response to -50 pA and -100 pA (n=28, N=9). ***D,*** 3D-plot of the two clusters obtained from the k-means algorithm for cholinergic/non-GABAergic neurons: EGFP^-^1 (grey) and EGFP^-^2 (black) (P12-P15, n=14, N= 6 and n=4, N=3, respectively); Parameters: AHP amplitude, delay of the 1^st^ spike in response to juxta threshold depolarization and maximal frequency. The two clusters obtained for P12-15 cholinergic/GABAergic neurons (green and dark green, see Fig. 5D) are superimposed. Insets in A,B and C,D show percentage of cells belonging to each cluster. ***E***, Silhouette coefficient plot for EGFP^-^1 and EGFP^-^2 clusters. ***F-H,*** Quantification of delay of the 1^st^ spike in response to juxta threshold depolarization, maximal frequency and AHP amplitude of GABAergic (green) and non-GABAergic (grey) cholinergic neurons from dataset at P2-5 and 4 clusters obtained from the k-means algorithm at P12-15 (EGFP^+^1, EGFP^+^2, EGFP^-^1, EGFP^-^2). ***I***, Representative membrane voltage responses to maximal depolarizing current pulses (black) and ***J***, corresponding voltage responses to the minimum current (rheobase) required to evoke an action potential (red) at expanded time scale for a P2-5 EGFP^-^ neuron and for two different P12-15 neurons belonging to the EGFP^-^1 or EGFP^-^2 cluster, as indicated. n=number of cells, N=number of mice. Mean ± SEM, *: P < 0.05; **: P < 0.01; ***P: < 0.001 (Extended Data Table 4-1). Download Figure 4-2, TIF file.

### Cholinergic/non-GABAergic neurons also constitute a single population

Typical examples of reconstructed cholinergic/non-GABAergic (double arrow) and cholinergic/GABAergic (arrows) neurons at P5 are shown in Extended Data Fig. 2-2*B*. Statistical analysis of Sholl parameters did not reveal significant differences of dendritic tree parameters at P0/1 between cholinergic/GABAergic and cholinergic/non-GABAergic cells (but this could be due to the sample size), but later, at P4/5, cholinergic/non-GABAergic cells developed somewhat differently (Extended Data Fig. 2-2*C*–*K*; Extended Data Table 2-1). The great majority of electrophysiological parameters were similar at P0/1 and at P4/5 (Extended Data Fig. 3-2; Extended Data Table 3-1). Using the same parameters for hierarchical cluster analysis (Fig. 4), we showed that P2/5 cholinergic/non-GABAergic neurons (EGFP^−^) also constituted a single population of early-firing neurons (Extended Data Fig. 4-2*A*,*F*–*J*). We did not observe significant differences of the parameters tested (maximal frequency of evoked spikes, delay of the fist spike in response to juxtathreshold depolarization, and AHP maximal amplitude) between cholinergic/GABAergic and cholinergic/non-GABAergic cells (Extended Data Fig. 4-2*B*,*F*–*H*; Extended Data Table 4-1). These results show that cholinergic/GABAergic and cholinergic/non-GABAergic neurons were both present at E18 in the SI/NBM region and constituted a single population of early-firing neurons throughout the first postnatal days (P2/5). Since ChAT^+^ EGFP^+^ and ChAT^+^ EGFP^−^ neurons shared similar electrophysiological characteristics, we extended our investigations to ChAT^+^ EGFP^+^ neurons only.

### Activation of GABA_A_Rs excites cholinergic/GABAergic neurons during the first postnatal week

To test the polarity of the GABA_A_R-mediated response of cholinergic/GABAergic neurons during the first postnatal week, we recorded the effects of the GABA_A_R agonist isoguvacine on their spontaneous activity in noninvasive, cell-attached, patch-clamp recordings. From P2 to P7, focal application of isoguvacine increased the frequency of ongoing spike activity to 2.65 times (*n* = 9; *p* = 0.003) at P2/3, to 2.52 times (*n* = 12; *p* = 0.001) at P4/5, and to 1.77 times (*n* = 16; *p* = 5 × 10^−5^) at P6/7 that of control (Fig. 5; Extended Data Table 5-1). In 4 out of 10 cells, we observed a dual excitatory–inhibitory effect of isoguvacine in some of the recordings, the inhibitory part resulting from the shunting effect of isoguvacine as described by Branchereau and colleagues (Branchereau et al., 2016). These results suggest that activation of GABA_A_R by afferent GABAergic synapses depolarize and activate postsynaptic cholinergic/GABAergic neurons throughout the first postnatal week.

10.1523/ENEURO.0538-23.2024.t5-1 Table 5-1 **Statistical analysis related to Figure 5.** Summary of statistical tests for Figure 5***C.*** 95% C.I. of diff - confidence interval for effect size, LCL-Lower Confidence Interval, UCL- Upper Confidence Interval. Download Table 5-1, DOCX file.

### Cholinergic/GABAergic neurons are spontaneously active and generate GABA_A_R- and AMPAR-mediated currents already at birth

**Figure 6.**
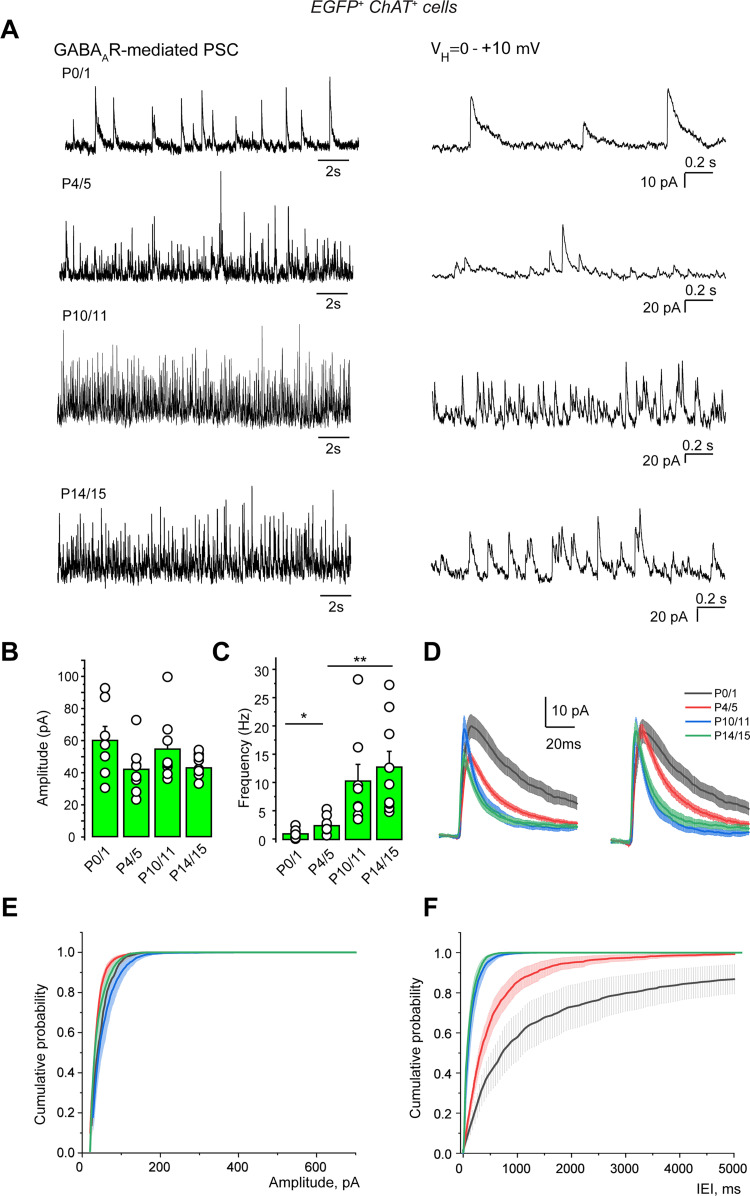
Development of the spontaneous GABA_A_R-mediated PSC recorded from cholinergic/GABAergic neurons (P0–15). ***A***, Left, Representative traces of spontaneous GABAergic PSCs (whole-cell voltage–clamp recordings, *V*_H_ = +10 mV) at the indicated ages. Right, Same recordings at an extended time scale. ***B***, Mean amplitude. ***C***, Mean frequency. ***D***, Averaged traces (original on the left, normalized to the peak amplitude on the right) of spontaneous GABAergic PSCs at the same ages as in ***A***. ***E***, Cumulative probability of spontaneous GABAergic PSC amplitude. ***F***, Cumulative probability of GABAergic PSCs interevent intervals (IEI; P0/1: *n* = 7, *N* = 5; P4/5: *n* = 9, *N* = 7; P10/11: *n* = 8, *N* = 4; P14/15: *n* = 9, *N* = 6). *n*, number of cells; *N*, number of mice. Mean ± SEM, *: *p* < 0.05; **: *p* < 0.01; ***: *p* < 0.001 (Extended Data Fig. 6-1).

**Figure 7.**
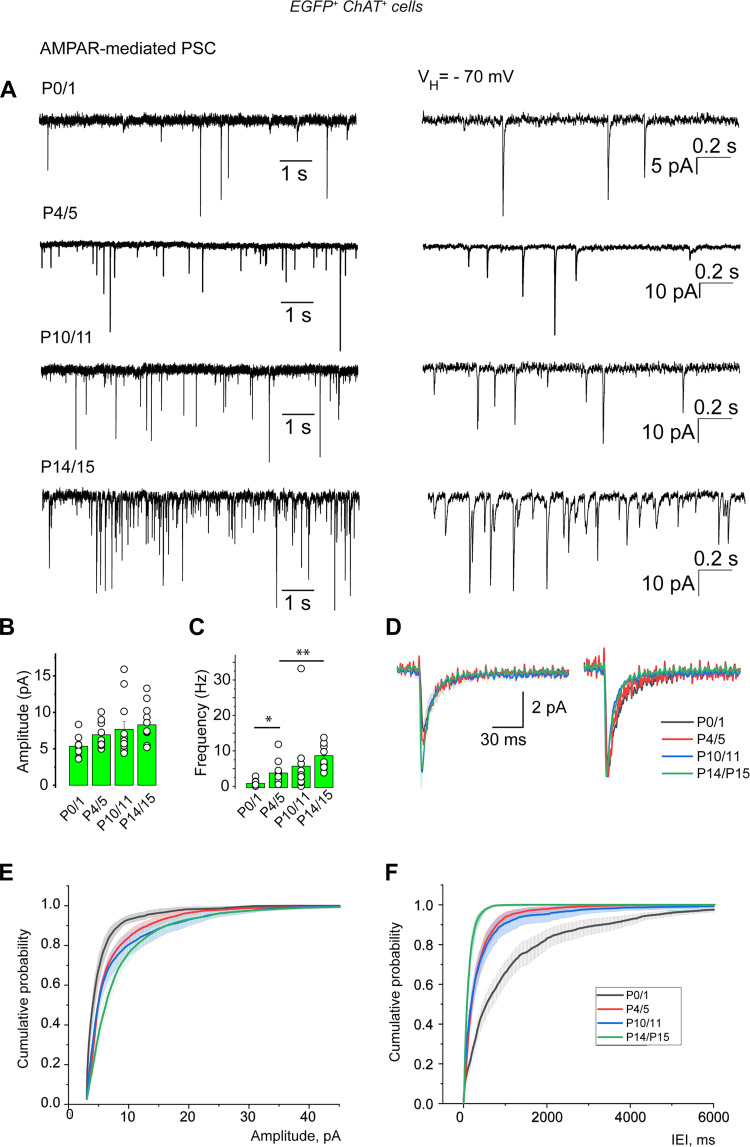
Development of the spontaneous AMPAR-mediated PSCs recorded from cholinergic/GABAergic neurons (P0–15). ***A***, Left, Representative traces of spontaneous AMPAR-mediated PSCs (whole-cell voltage–clamp recordings, *V*_H_ = −70 mV) at the indicated ages. Right, Same recordings at an extended time scale. ***B***, Mean amplitude. ***C***, Mean frequency. ***D***, Averaged traces (original on the left, normalized to the peak amplitude on the right) of spontaneous AMPAR-mediated PSCs at the same ages as in ***A***. ***E***, Cumulative probability of spontaneous glutamatergic PSCs amplitude. ***F***, Cumulative probability of IEI. P0/1: *n* = 9, *N* = 6; P4/5: *n* = 12, *N* = 6; P10/11: *n* = 12, *N* = 7; P14/15: *n* = 10, *N* = 4. *n*, number of cells; *N*, number of mice. Mean ± SEM. *p < 0.05; **p < 0.01; ***p < 0.001 (Extended Data Fig. 7-1).

Spontaneous postsynaptic outward currents (*V*_H_ = +10 mV), sensitive to picrotoxin (50 µM), were already present at birth. These GABA_A_R-mediated PSCs had a mean amplitude of 60.0 ± 8.7 pA and a mean frequency of 1.0 ± 0.3 Hz at P0/1 (*n* = 7; Fig. 6; Extended Data Table 6-1). Spontaneous postsynaptic inward currents (*V*_H_ = −70 mV) sensitive to CNQX (10 µM) were also already present at birth. These AMPAR-mediated PSCs had a mean amplitude of 5.4 ± 1.5 pA and a mean frequency of 1.2 ± 0.3 Hz at P0 (*n* = 9; Fig. 7; Extended Data Table 7-1). The frequencies of these spontaneous postsynaptic outward and inward currents significantly increased during the first postnatal week.

10.1523/ENEURO.0538-23.2024.t6-1 Table 6-1 **Statistical analysis related to Figure 6.** Summary of statistical tests for Figure 6 *B,C.* 95% C.I. of diff - confidence interval for effect size. Download Table 6-1, DOCX file.

10.1523/ENEURO.0538-23.2024.t7-1 Table 7-1 **Statistical analysis related to Figure 7.** Summary of statistical tests for Figure 7 *B,C.* 95% C.I. of diff - confidence interval for effect size. Download Table 7-1, DOCX file.

**Figure 8.**
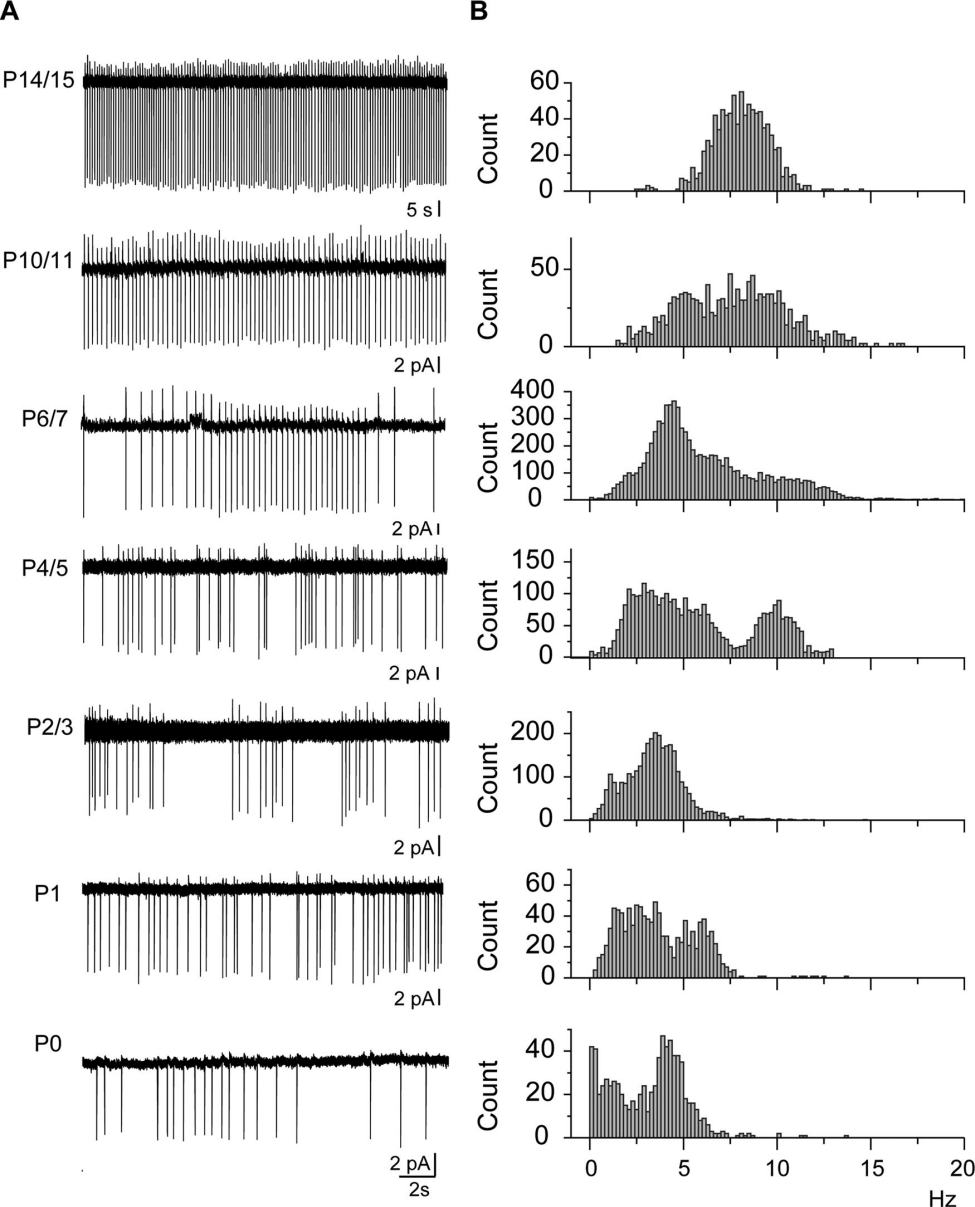
Patterns of ongoing activity of cholinergic/GABAergic neurons (P0–P15). ***A***, Representative cell-attached recordings at the indicated ages. ***B***, Pooled instantaneous frequency distributions of ongoing activities recorded as in ***A***: (P0: *n* = 7, *N* = 3, 800 events; P1: *n* = 6, *N* = 3, 1,070 events; P2/3: *n* = 12, *N* = 3, 3,211 events; P4/5: *n* = 8, *N* = 3, 3,660 events; P6/7: *n* = 10, *N* = 4, 10,015 events; P10/11: *n* = 10, *N* = 6, 1,323 events; P14/15: *n* = 5, *N* = 3, 1,332 events). *n*, number of cells; *N*, number of mice.

These results show that GABA_A_ and AMPA synapses afferent to cholinergic/GABAergic neurons were present and spontaneously active already at birth. Until GABA polarity shift occurred, both GABA_A_R- and AMPAR-mediated PSCs had a depolarizing action (Fig. 5). This suggests that during the first postnatal week, both GABAergic and glutamatergic afferents excite cholinergic/GABAergic neurons which are all of the early-firing type (Fig. 4*A*,*B*; Extended Data Fig. 4-2*A*,*B*). These excitations should sustain their spontaneous activity. Noninvasive cell-attached recordings (so as not to change the intracellular concentration of chloride ions) revealed that cholinergic/GABAergic neurons were spontaneously active already at birth with a mean frequency of 3.15 ± 0.07 Hz (*n* = 800 events, seven cells). The pattern of activity was irregular with bursting periods up to P6/7, as attested by the presence of frequencies up to 15 Hz and over (Fig. 8).

### During the second postnatal week GABA becomes inhibitory and cholinergic neurons separate into two populations endowed with different electrical properties

We then studied how GABAergic and non-GABAergic cholinergic neurons developed in parallel with GABA polarity shift. During the second postnatal week, three of the morphological parameters studied exponentially increased after their transient decrease (area under the curve, total dendritic length, and critical value). For all the other parameters, we did not observe significant changes after P4/5 (but this could be due to the sample size; Fig. 2*C–H*; Extended Data Table 2-1). When comparing morphological parameters of cholinergic/GABAergic neurons with cholinergic/non-GABAergic neurons, we did not observe significant differences at P14/15, despite the somewhat different development of some of the parameters of cholinergic/non-GABAergic neurons (Extended Data Fig. 2-2*C*–*K*; Extended Data Table 2-1). Similarly, we did not observe significant differences of their electrophysiological parameters at P14/15, despite their differences of AP and AHP amplitude at P10/11 and AP half-width at P4/5 and P10/P11 (Extended Data Fig. 3-2; Extended Data Table 3-1).

Isoguvacine application significantly decreased spike frequency at P14/15 (two times that of control; *n* = 7; *p* = 0.003; Fig. 5; Extended Data Table 5-1). GABA polarity shift occurred from P8/9 in cholinergic/GABAergic neurons since from this age isoguvacine either had no effect or significantly decreased spike frequency compared with the control. The frequency (but not the amplitude) of spontaneous GABA_A_R- and AMPAR-mediated PSCs considerably increased from P4/5 to P14/15: from 2.5 ± 0.5 Hz (*n* = 9) to 13.1 ± 2.9 Hz (*n* = 9; *p* = 0.004) and from 4.2 ± 0.9 Hz (*n* = 12) to 9.2 ± 1.2 Hz, (*n* = 10; *p* = 0.004), respectively (Figs. 6, 7).

Earlier studies have suggested the presence of at least two populations of cholinergic neurons in the adult mouse forebrain (Unal et al., 2012; Laszlovszky et al., 2020). We observed a similar dichotomy at P12/15. For example, maximal frequency, AHP, and sag amplitudes were highly heterogeneous for P12/15 cholinergic/GABAergic neurons (Figs. 3*B*,*E*,*I*, 4*F*,*G*). In addition, the mean delay of the first spike in response to a juxtathreshold depolarization (rheobase) significantly increased from 239.3 ± 2.0 ms at P2/5 (*n* = 25) to 407.7 ± 48.8 ms at P12/15 (*n* = 30; *p* = 0.006). Hierarchical and *k*-means clustering analyses identified two groups of P12/15 cholinergic/GABAergic neurons (EGFP^+^, *n* = 30): EGFP^+^1 (light green; *n* = 18; silhouette index, 0.72) and EGFP^+^2 (dark green; *n* = 12; silhouette index, 0.65; Fig. 4*C*–*E*; Extended Data Table 4-1). Comparison of electrophysiological parameters between the two populations of P12/15 cholinergic/GABAergic neurons populations (EGFP^+^1 and EGFP^+^2) revealed significant differences for delay of the fist spike, maximal frequency, and AHP maximal amplitude (Fig. 4*F–J*; Extended Data Table 4-1). We named the EGFP^+^1 population early-firing and the EGFP^+^2 population late-firing.

Similar clustering analyses also revealed two populations of P12/15 cholinergic/non-GABAergic (EGFP^−^) neurons (*n* = 18, *N* = 9), the early-firing (EGFP^−^1; gray; *n* = 14; silhouette index, 0.68) and late-firing (EGFP^−^2; black; *n* = 4; silhouette index = 0.64) populations (Extended Data Fig. 4-2*C*–*E*). We did not observe significant differences between parameters tested (maximal frequency of evoked spikes, delay of the fist spike in response to juxtathreshold depolarization, and AHP maximal amplitude) between EGFP^+^1 versus EGFP^−^1 and EGFP^+^2 versus EGFP^−^2 (Extended Data Fig. 4-2*D*,*F*–*J*; Extended Data Table 4-1).

Collectively, these results show that the major changes in dendritic trees and membrane parameters of all cholinergic neurons principally occurred during the first postnatal week. Extrinsic parameters such as afferent connections activity was present from P0 and considerably increased during the second postnatal week. They also highlight the low impact of GABAergic cotransmission on the development and types of functional cholinergic populations. After the first postnatal week, in parallel to GABA polarity shift, with the increase of active glutamatergic and GABAergic connections and the regularization of spontaneous in vitro activity, cholinergic GABAergic or non-GABAergic neurons separate into two distinct populations according to intrinsic electrophysiological parameters. The new population, the late-firing neurons, represented 40% of the P12/15 cholinergic neurons. They were less excitable than early-firing neurons since they responded with a longer delay to juxtathreshold depolarizations and with a lower maximal frequency to large depolarizations (Fig. 4*F*,*G*,*I*,*J*; Extended Data Fig. 4-2*F*,*G*,*I*,*J*). They also displayed a large AHP maximal amplitude (Fig. 4*H*; Extended Data Fig. 4-2*H*). These two populations corresponded to the two populations previously described in adult rodents in vitro and in vivo (Unal et al., 2012; Laszlovszky et al., 2020).

## Discussion

From perinatal days up to the end of the first postnatal week, when GABA_A_R current polarity shift has not yet occurred, SI/NBM cholinergic neurons, whether they have coexpressed or still express GABAergic markers, belong to a single homogenous population of developing, spontaneously active, bursting, and early-firing neurons, connected to and depolarized by both their glutamatergic and GABAergic afferents. At that stage, during the first postnatal days, the only heterogeneity of SI/NBM cholinergic neurons concerned their ability to corelease GABA. During these early postnatal days, between P2/3 and P8/9, the significant decrease in some of the morphological parameters of SI/NBM cholinergic neurons (area under the Sholl curve, total dendritic length, critical value, and number of dendritic nodes) probably reflects dendrite pruning. This process of dendrite elimination that initially forms in abundance occurs during the early stages of normal development of neuronal networks. It is essential to sculpt the mature nervous system of both vertebrates and invertebrates (Konur and Ghosh, 2005).

The excitatory effect of the GABA_A_R agonist isoguvacine until P8/9 suggests that GABAR-mediated transmission is depolarizing and excitatory during the first postnatal week. Bath applied isoguvacine could, however, have an indirect excitatory effect on SI/NBM neurons at P5 via the release of glutamate by glial cells (excited by isoguvacine) or via the inhibition of excitatory interneurons (disinhibition). But glia is much less developed in very young brains than later. Although the developmental shift in GABA action has been widely demonstrated in vitro (for review, see Ben Ari et al., 2007), results in vivo in anaesthetized animals (Kirmse et al., 2015) have challenged this finding. But experiments carried out in nonanesthetized pups (Murata and Colonnese, 2020) showed a clear excitatory effect of GABA interneurons in vivo in young rodents. Persistent shift of GABA polarity from depolarizing to hyperpolarizing mode is due to the reduction in the intracellular chloride concentration and is a critical event for early brain circuits’ development and their orchestrated activity-dependent formation.

SI/NBM cholinergic neurons generated AMPAR-mediated and GABA_A_R-mediated spontaneous currents already at birth, with a frequency which increased during the first postnatal week. These inputs may originate, as in the adult, from locally arborizing glutamatergic and GABAergic neurons which form complex networks with cholinergic neurons (Zaborszky and Duque, 2000; Gritti et al., 2006; McKenna et al., 2021). Double immunolabeling for ChAT and the vesicular glutamate transporters (Vglut) in the adult rat SI showed synapses between Vglut1 or Vglut2 boutons and cholinergic dendrites and occasionally between Vglut2 boutons and cholinergic cell bodies (Hur et al., 2009). Glutamatergic inputs can also arise from cortical areas since the anterograde transport of *Phaseolus vulgaris* leucoagglutinin (PHA-L) combined with AChE histochemistry or ChAT immunocytochemistry revealed rich PHA-L-labeled projections to discrete parts of the BF cholinergic system essentially originating from all adult prefrontal areas investigated (Gaykema et al., 1991).

GABA_A_R-mediated spontaneous currents were present at birth and then increased in frequency. Afferent GABAergic terminals were likely to arise from both local and extrinsic afferent sources. BF GABAergic cells are about twice as numerous as BF cholinergic cells (Gritti et al., 1993). GABAergic (GAD^+^) varicosities were identified in the immediate vicinity of biocytin-filled soma and dendrites of electrophysiologically identified cholinergic cells in the adult guinea pig NBM (Khateb et al., 1998) and GABAergic axons synapse onto adult rat cholinergic neurons (Záborszky et al., 1986). Amygdala, striatum, and accumbens also send GABAergic projections to adult BF cholinergic neurons (Záborszky et al., 1986; Záborsky and Cullinan, 1992; Paré and Smith, 1994; Gielow and Zaborszky, 2017). GABA_A_R-mediated spontaneous PSCs were excitatory until the end of the first postnatal week.

Interestingly, P0/5 SI/NBM cholinergic neurons received glutamatergic and GABAergic inputs which were both excitatory, and they responded with a short delay to juxta- and suprathreshold depolarizations. This, combined to a high membrane resistance, a small amplitude AHP, and a large sag suggesting the presence of a pacemaker H current, allowed P0/P5 cholinergic neurons to spontaneously fire. Moreover, P0/P5 cholinergic neurons tended to spontaneously burst. This bursting activity is likely to propagate to the cortex where cholinergic terminals are already present. All these characteristics strongly suggest that during the first postnatal days, SI/NBM cholinergic neurons can spontaneously release ACh or ACh and GABA in the developing cortical layers allowing them to fulfill their role in cortical development during the first postnatal days. Coreleased GABA, which is probably depolarizing/excitatory to cortical cells during early postnatal days, should also play a role in cortical development (Represa and Ben-Ari, 2005).

During the second postnatal week, a second population of SI/NBM cholinergic neurons appeared, the late-firing neurons, which fired with a longer delay in response to subthreshold depolarizations, displaying a lower maximal frequency due to a large-amplitude AHP. The same two cholinergic populations have been described in the adult rodent BF, in vitro and in vivo (Unal et al., 2012; Laszlovszky et al., 2020). We show here that the classification of SI/NBM cholinergic neurons in early or late-firing populations was uncorrelated to the coexpression of GABA release markers (Kosaka et al., 1988; Tkatch et al., 1998; Saunders et al., 2015; Granger et al., 2016).

The two electrophysiologically identifiable types of BF cholinergic neurons, the early- and late-firing neurons, may fulfill different functions in the adult in vivo (Laszlovszky et al., 2020). The in vivo BF_BURST_ (bursting firing) neurons (70%), corresponding to the in vitro early-firing neurons, generated bursts with a strong synchrony with each other and auditory cortical oscillations, preferentially after behavioral reinforcement, water reward, or air-puff punishment, suggesting that they may have a strong impact on cortical processing. In contrast, the in vivo BF_REG_ (regular firing) neurons (30%), corresponding to the in vitro late-firing neurons, were nonbursting neurons with shorter spikes and a bigger AHP. Coupling between regular firing neurons and the auditory cortical neurons was strongest before mice made successful hits, thus predicting behavioral performance. The proportion of each subpopulation varied between anterior and posterior BF. As the anterior and posterior BF have different projection targets (Rye et al., 1984; Unal et al., 2012), distinct brain regions receive different proportions of bursting cholinergic input.

The functional diversity of BF cholinergic neurons may result from the multiplicity of embryonic origins (medial ganglionic eminence, preoptic area, septal neuroepithelium; Magno et al., 2017), ventral area of the pallial telencephalon (pallium; Pombero et al., 2011), and/or combinatorial gene expression including a number of transcription factors during early developmental stages (see review in Allaway and Machold, 2017; Ahmed et al., 2019) and/or from their birth date (Allaway et al., 2020). The expression or not of GABA corelease markers is the only functional diversity present in the perinatal period. Between P0 and P7, all SI/NBM cholinergic neurons have properties (highly excitable, excited by both glutamatergic and GABAergic afferents) that give them the capacity to provide ACh or ACh and GABA to the developing cortex. It is only during the second postnatal week, in parallel to GABA polarity shift, that two functional populations of SI/NBM cholinergic neurons are present and fulfill two different roles in cortical processing. The present characterization of the early postnatal activity of basal cholinergic neurons allows understanding the crucial role of ACh in the early development of cortical networks. Furthermore, the transition of NB/SI cholinergic neurons from a single population of early responding neurons, activated by both GABA and glutamate during the postnatal period, to two populations of neurons with more complex properties from P15 onward is an example of how neurons in a structure shift from a developmental role to a functional role.

## Conclusion

We propose that the early development of a single and homogeneous population of highly excitable SI/NBM cholinergic neurons (GABAergic or not), together with their activation by both their glutamatergic and GABAergic afferents, allows the release of ACh and GABA in the developing sensorimotor cortical layers already during the first postnatal week (Gaykema et al., 1991; Saunders et al., 2015; Zaborszky et al., 2015; Granger et al., 2016; Gielow and Zaborszky, 2017). This would explain their early determinant role in the development of cortical neurons and networks (Hohmann and Berger-Sweeney, 1998). During the second postnatal week, in parallel with the GABA polarity shift, SI/NBM cholinergic neurons differentiate into two distinct populations that exert different functional roles in adults (Laszlovszky et al., 2020).

## Synthesis

Reviewing Editor: Selva Baltan, Oregon Health &Science University Department of Surgery Decisions are customarily a result of the Reviewing Editor and the peer reviewers coming together and discussing their recommendations until a consensus is reached. When revisions are invited, a fact-based synthesis statement explaining their decision and outlining what is needed to prepare a revision will be listed below. The following reviewer(s) agreed to reveal their identity: Heng Zhou.

Dear Authors,

Thank you for submitting your study to eNeuro.

The reviewers have provided extensive feedback for your revision of the manuscript. Please address these diligently by;

1) Addressing the concerns about power analysis and statistical analysis to improve the reproducibility and rigor of the study.

2) Revising and using appropriate scientific terminology while making comparisons.

3) Emphasizing the shortcomings and alternative interpretations in the discussion

4)Compiling and providing clarification for the raised issue for figures.

Thank you.

## Author Response

Dear Editor and Reviewers We would like to thank the reviewers for their in-depth analysis of our results. They helped us greatly improve our manuscript. We have carefully addressed all points and made changes to the manuscript accordingly. Please find our responses below in blue.

Response to the Editor The reviewers have provided extensive feedback for your revision of the manuscript. Please address these diligently by:

1) Addressing the concerns about power analysis and statistical analysis to improve the reproducibility and rigor of the study.

Response: We performed statistical power analysis for all cases when p superior 0.05 as asked by the Reviewer.

We reran all our statistical analyses, included confidence intervals (using alpha level 0.05 stating that we are 95% confident that [population parameter] is between [lower bound, upper bound]) and included effect size. We performed silhouette analysis as asked by Reviewer 2 for P14-15 results in the New figure 4 and the New Extended data Figure 4-2. This confirmed the existence of two neuronal populations at P14-15.

These analyses are explained in the Methods (section Statistics) and are all included in Extended data Tables.

2) Revising and using appropriate scientific terminology while making comparisons.

Response: When making comparisons between two sets pf data (as in New Extended data figures 2-2 we wrote for example page 10 line 225: "Sholl parameters did not reveal significant differences of dendritic tree parameters at P0/1 between cholinergic/GABAergic and cholinergic/non-GABAergic cells (but this could be due to sample size)".

3) Emphasizing the shortcomings and alternative interpretations in the discussion Response: This refers to points 3 &4 of Reviewer 1 and to points 1,2,3 &4 of Reviewer 2. We answered all these points and modified the MS accordingly.

4) Compiling and providing clarification for the raised issue for figures. Thank you.

Response: These are the comments from R1 point 2 and from R2 points 9 to 13. We modified our figures accordingly.

New Figure 1: is a merge of old figures 1 and 2A as asked by Reviewers 1 &2.

New Figure 2: is old figure 3 but the panels were reorganized, and a new part A was added as asked by Reviewer 2.

New extended data Figure 2-2 is old extended data figure 3-2 but the panels were reorganized, and a new part A was added as asked by Reviewer 1 point 2.

New Figure 3: is a merge of old figures 2B and 4 with a new part A was added as asked by Reviewer 2.

- Response to Reviewer 1 This study is to investigate the morphological and electrophysiological properties of cholinergic neuron during a key development window. Overall, their experiments were nicely performed, and their results are rigid. However, I think the significance of this paper is a bit weak because of the lack of crucial description, which should be clarified before publication.

1. The expression proportion of ChAT/GFP+ and ChAT/GFP- cell from E18 to P15, change or not？ Response: We indeed tried to count the number of ChAT+/GFP+ and ChAT+/GFP- cells in NB/SI as a function of age to assess whether their proportion changed with age. This is extremely delicate work because, as we count in slices, it is easy to count twice the same cell present in adjacent slices. We also had to delineate NBM and SI regions in the same way at all ages, regions which are not well demarcated. We therefore decided not to include any of these results.

2. A description of Figure 1 needs to be included in the results.

Response: We included the description for all parts of the New figure 1 lines 191 to 201.

Figure 1 and Figure 2 also be merged into one figure, if possible, can you provide a tracer of biotin filled cell during patch-clamp for ChAT/GFP- cell indication? Response: In agreement with Reviewers 1 and 2 we reorganized figures 1 to 4:

New Figure 1: is a merge of old figures 1 and 2A as asked by Reviewers 1 &2.

New Figure 2: is old figure 3 but the panels were reorganized, and a new part A was added as asked by Reviewer 2.

New extended data Figure 2-2 is old extended data figure 3-2 but the panels were reorganized, and a new part A was added as asked by Reviewer 1 point 2.

New Figure 3: is a merge of old figures 2B and 4 with a new part A was added as asked by Reviewer 2.

3. Why did the area under the curve, cell ending radius, total dendritic length, and critical radius exponentially increase from E18 to P3 or P5 but decrease at P6/7 and p8/9, while the frequency of firing increased.

Response: Based on our new extended statistical analyses (see Methods), 4 morphological parameters of GFP+ cholinergic neurons significantly and exponentially decreased from P2/3 to P8/9 after an exponential increase or a plateau: area under the curve, total dendritic length, critical value, and number of dendritic nodes (see New Figure 2 and Extended data table 2). We reorganized the panels of the new Figure 2 compared to previous Figure 3, to show these parameters close to each other. We discussed page 14 lines 332-337 that these synchronous decreases probably reflect pruning of dendrites during that period.

The firing frequency (and pattern) of a neuron depends on its intrinsic membrane properties as well as the number and activity of its excitatory afferents. To the best of our knowledge, it is not strictly correlated with dendritic surface area. Moreover, pruning can be activity dependent.

4. The order of the figures should be consistent with the resulting narrative.

Response: We have made sure that this is now the case.

5. This paper explores the morphological and electrophysiological changes in the development of acetylcholine neurons, and its practical significance needs to be further clarified.

Response: We added a new part at the end of the discussion page 17 lines 407-412: "The present characterization of the early postnatal activity of basal cholinergic neurons allows understanding the crucial role of acetylcholine on the early development of cortical networks. Furthermore, the transition of NB/SI cholinergic neurons from a single population of early responding neurons, activated by both GABA and glutamate during the postnatal period, to two populations of neurons with more complex properties from P15 onwards, is an example of how neurons in a structure shift from a developmental role to a functional role." Response to Reviewer 2 Fundamental issues 1. The increased firing in response to isoguvacine as an evidence for excitatory GABA is not very convincing, since it may be an indirect circuit effect, involving for instance disinhibition or release of glutamate by glial cells. The authors should accordingly either discuss and mitigate their conclusions or clarify this point with additional experiments allowing to distinguish between GABAergic and glutamatergic inputs responsible for the increased firing (eg monosynaptic GABAergic and glutamatergic postsynaptic responses in gramicidin-perforated patch).

Response: It is of course possible that isoguvacine has an indirect effect such as the direct excitation of glial cells and their release of glutamate in very young brains, in the absence of direct excitation of neurons. But then 10 days later isoguvacine would directly inhibit both neurons and glial cells? Regarding the first hypothesis, it is important to take into account that Glia is poorly developed at a very early stage where its activity is discussed. We discussed these possible indirect effects of isoguvacine page 14 lines 338-342: "Bath applied Isoguvacine could have an indirect excitatory effect on SI/NBM neurons at P5 via the release of glutamate by glial cells (excited by isoguvacine) or via the inhibition of excitatory interneurons (disinhibition). However, glia is much less developed in very young brains than later." 2. Besides, GABAergic transmission, as well as prolonged application (here in the bath) of isoguvacine, is expected to result in shunting inhibition, irrespective of an initially inhibitory or excitatory effect. This point should be at least discussed, if not addressed experimentally with electrophysiological recordings, for example using whole cell or gramicidin-perforated patch. Recording the effects of GABAergic blockers on spontaneous activity may help clarify the potential expression of a globally inhibitory effect of GABAergic transmission on network activity.

Response: The shunting effect is due to an increase in membrane conductance owing to the synchronous opening of many GABAA channels (or other channels). It mainly depends on the driving force for chloride ions, the chloride conductance, the neuronal input resistance, and the spatial arrangement of GABA synapses (whether they are packed or not).

Shunting inhibition is always a possibility, but in the perinatal period the developmental aspect must be considered. In immature neurons, the shunting action is not always observed because of the lower number of GABAA channels at that stage (see figure 7A). At young stages, when present, it results in dual excitatory (GABAAR-mediated) and inhibitory (shunting) components. The predominance of shunting inhibition is mainly observed with high frequency stimulation of GABA afferents (due to chloride accumulation and the change of ECl) (Branchereau et al., 2016).

In the case of bath applied isoguvacine, a dual excitatory-inhibitory response to isoguvacine, due to the duration of bath application, was sometimes recorded but never an inhibition alone before P7. Then after P12, when GABAAR-mediated responses are inhibitory, a superimposed inhibitory effect due to synaptic transmission and shunting, are present.

We discussed these points in the Results page 11 lines 247- 249: "In 4 out of 10 cells, we observed a dual excitatory-inhibitory effect of isoguvacine in some of the recordings, the inhibitory part resulting from the shunting effect of isoguvacine as described by Branchereau and coll (Branchereau et al., 2016)." 3. In general, unlike stated several times in the text (eg p.10: "during foetal early postnatal periods, GABA is the primary depolarizing/excitatory neurotransmitter"), the question of depolarizing/excitatory GABAergic transmission, whether during development or in pathological conditions, is controversial in vivo. This assertion should be mitigated throughout the text and additional references should be included to better reflect the current state of knowledge on early GABAergic transmission.

Response: The developmental shift of the GABAAR-mediated transmission is now a solid accepted observation in vitro in brain slices (from rodents to humans included). A library of papers by the groups of Ben-Ari, Fukuda, Luhmann, Kriegstein and many others have amply shown that. Considering the in vivo issue, there are 2 papers that challenged the depolarizing effect of GABAAR activation in pups in vivo (but validating them in vitro). These results were based on in vivo recordings in anesthetized animals. Anesthetics are well-known to produce strong hyperpolarizing action of GABA and this is in fact one of the mechanisms underlying their anesthetic actions. This has now been shown to be the case in the work of Murata &Colonnese showing that indeed in non-anesthetized rodents GABA including synaptically released GABA is excitatory on hippocampal neurons.

We added two references and a paragraph on this subject in the discussion p14-15 lines 342-348: "Although the developmental shift in GABA action has been widely demonstrated in vitro (for review see (Ben Ari et al., 2007)), results in vivo in anaesthetized animals (Kirmse et al., 2015) have challenged this finding. But experiments carried out in non-anesthetized pups (Murata and Colonnese, 2020) showed a clear excitatory effect of GABA interneurons in vivo in young rodents. Persistent shift of GABA polarity from depolarizing to hyperpolarizing mode is due to the reduction in the intracellular chloride concentration and is a critical event for early brain circuits' development and their orchestrated activity-dependent formation. " Kirmse K, Kummer M, Kovalchuk Y, Witte OW, Garaschuk O, Holthoff K. GABA depolarizes immature neurons and inhibits network activity in the neonatal neocortex in vivo. Nat Commun. 2015;6:7750. doi: 10.1038/ncomms8750 Murata Y, Colonnese MT. GABAergic interneurons excite neonatal hippocampus in vivo. Sci Adv. 2020;6(24): eaba1430. doi: 10.1126/sciadv.aba1430. eCollection 2020 Jun.PMID: 32582852 4a. The authors claim that two distinct types of cells can be identified from clustering analysis, but the evidence they provide to support their claim is not very convincing. The comparisons made in Fig.5G-I are rather trivial (if you compare the first half to the second half of a distribution, they will tend to be statistically different by construction).

Response: The comparison made in Fig.5G-I and Extended Fig.5-2 G-I (new Figs 4 F,G and 4-2 F,G) was not a comparison between first and second parts of distributions. It was strictly based on cluster membership obtained from k-means analysis.

4b. In Supplementary Fig 5.2, it looks like the number of values is really low to conclude for distinct clusters in GABA- cells (cf D: only 3 AGFP-2 cells?). Therefore, the authors should either provide appropriate statistics (e.g. Silhouette coefficient, Davies-Bouldin index, Calinski-Harabasz index) to validate their claim of statistically meaningful distinct clusters, or provide additional evidence, such as for instance the histogram distributions of AHP, AP latency (which actually appears as the most relevant) and AP max frequency presented in Fig 5G-I to evaluate the potential expression of bimodal distributions.

Response: For EGFP-2 cluster there are 4 cells, not 3.

We performed silhouette coefficient to validate (or not) the claim of two populations at P15. The result validated the existence of two populations, and this analysis is now present in the new Fig. 4 and the new Extended data Fig. 4-2 (former Figures 5 and extended data Fig. 5-2) and in the Methods page 8 lines 183-187.

5. For all results in which the conclusions are that there is no statistically significant difference, power analysis should systematically be provided. Otherwise, the authors should indicate that in absence of observed statistically significant difference, they cannot make any conclusion regarding whether the distributions of values are different or similar, which would definitely greatly reduce the significance of their study.

Response: In order not to assert that there is no significant difference where there could be a significant one, we performed statistical power analysis for all cases when p superior 0.05 as asked by the Reviewer. Moreover, to provide the readers with an estimate of the strength of our conclusions for each dataset, we have now included in each Extended data Table, confidence interval and the observed effect size.

Nevertheless, there is always the possibility that when a difference does exist, the statistical test is not able to identify it, Therefore, when making comparisons (as in New Extended data figures 2-2), in the text we wrote:

1) page 10, line 225: "Statistical analysis of Sholl parameters did not reveal significant differences of dendritic tree parameters at P0/1 between cholinergic/GABAergic and cholinergic/non-GABAergic cells (but this could be due to sample size)";

2)page 12, line 277: "For all the other parameters, we did not observe significant changes after P4/5 (but this could be due to sample size)".

Specific issues and clarifications required:

6. Some more detailed explanations should be provided to describe and justify why the use of Lhx6-iCre+/-;RCE-EGFP+/- mice was particularly suited to seperately record and label in the SI/NBM region.

Response: We added in the Methods page 4 lines 83-86: "We used slices from Lhx6-iCre+/-;RCE-EGFP+/- mice, because EGFP expression allows characterizing GABA/ACh neurons after posthoc ChAT immunocytochemistry. EGFP expression also allows delineating the border of globus pallidus (GP) since all GP cells are EGFP+ and fluorescent. This is particularly important in very young animals. " 7a. Throughout the results, all the parameters that have been quantifed need to be described: what is the "number of crossing intersection" (apparently not the number of nodes), cell ending radius (the distance between the soma and its most distant terminal?) critical radius (the radius of what exactly?), "number of dendritic trunks" (is that the number of dendrites leaving the soma?)... For clarity, this could be illustrated in a figure with arrows pointing at some examples of each on an individual cell.

Response: As asked by the referee, we added this figure as part A of the New Fig. 2 to clearly explain by this figure and its corresponding legend the parameters of the Sholl analysis (cell ending radius, critical radius, critical value...) shown in New Figure 2 and New Extended Figure 2-2.

We renamed dendritic trunks primary dendrites (line 207-208).

7b. The same holds for electrophysiological parameters: how were exactly determined spike threshold potential, amplitude, AHP, sag...? This may for instance clarify why the "sag" is described as decreasing with age while on the traces is apparetly increases from E18 to P7. Here also, this could be illustrated in a figure with arrows pointing at some examples of each on an individual trace.

Response: As asked by the referee, we added this figure to part A of the New Fig. 3 to clearly explain by this figure and its corresponding legend how we measured AP and sag parameters from an individual trace.

8. In the descriptive and comparison statistics, the number of independent measures should always be indicated (for instance, was 9.5 ± 1.9 Hz the mean maximal frequency of 13 (cells) or of 4 (mice) values?), and statistics actually provided for the number of mice (even if in addition to the number of cells pooled accross animals).

Response: The mean indicated in the text is always the mean of measures in individual cells (and not the mean of the mean in each animal). We suppressed the number of animals in the text but left it in the Figure legends and Tables. We added this clarification in the Methods page 7 lines 167-169.

9. The organization of Figure 2 and 3 should be revised to avoid overlap and redundancy. In Fig3, left and right plots should be superimposed for clarity and to make the figure more compact. Fig.2A is an illustration of what is quantified in Fig.3 (and Fig.2A and 3 are actually referred to in the text before Fig.2B), and Fig.2B is redundant with Fig.4A (and these actually referred to together in the first paragraph of p.8).

Response: In agreement with Reviewers 1 and 2 we reorganized figures 1 to 4 New Figure 1: is a merge of old figures 1 and 2A as asked by Reviewers 1 &2.

New Figure 2: is old figure 3 but the panels were reorganized, and a new part A was added as asked by Reviewer 2.

New extended data Figure 2-2 is old extended data figure 3-2 but the panels were reorganized, and a new part A was added as asked by Reviewer 1 point 2.

New Figure 3: is a merge of old figures 2B and 4 with a new part A was added as asked by Reviewer 2.

In new figure 2 and 3 we could not superimpose left and right panels as suggested by Reviewer 2 because the right panels are semilogarithmic and when we tried to merge both panels with two different scales it gave a messy figure.

In the same line, Fig.4 is partially redundant and partially contradictory (eg time course of AP at E18) with Fig.2B.

Response: Considering electrophysiological recordings, we now paid attention to choose traces that match the mean observations (amplitude of spikes at E18, rebound at P0). For the sag, the misunderstanding came from the fact that we illustrated sags in response to different current steps amplitudes as said in the legend (from -40 pA at E18 to -100 pA at P7). We have now illustrated sags in response to -40 and -100 pA at all ages (part A New figure 3).

In Suppl Fig 3.2, shouldn't the results from EGFP+ be exactly the same as in Fig.3 (with which this is supposed to be redundant)? And in Suppl Fig 4.2, shouldn't the results from EGFP+ be exactly the same as in Fig.4 (with which this is supposed to be redundant)? The authors should clarify these apparent contradictions and combine the figures to avoid redundancy as much as possible.

Response: We carefully checked. The results are the same in old figure 3 and Extended data fig 3-2 and in old figure 4 and Extended data figure 4-2. The difference is in the ages displayed: in extended data figures all histograms begin at P0/1 compared to E18 in main figures.

We have not seen how to change the figure to avoid redundancy since we want to compare data between EGFP+ and EGFP- cholinergic neurons during development.

10. In Fig3, what is the physiological relevance of "the area under the curve", compared for instance to the total number of "crossing intersections"? If the authors meant to provide synthetic statistics about their spatial repartition (distance from soma), wouldn't it make more sense to provide the distance of the peak and some characteristics of the decay with distance? Response: If we understand correctly, Reviewer 2 thinks that calculating the areas under the curves does not add information compared to the plots of the number of intersections versus distance from the soma. He suggests analyzing the distance between the peak of the curve and the soma and the decay of the curve with distance.

Area under the Sholl curve provides a measure of dendritic arbor complexity and therefore a useful single metric to compare overall Sholl profiles. It is commonly used (see for example Wang et al. 2018, Wilson et al 2017, Binley et al 2014, Pallotto 2012).

Distance of the peak from the soma i.e. the critical radius, as a function of age was present in old figure 3c and old Extended figure 3-2c. It is still present in New figure 2D and New Extended data figure 2-2F. We fitted the decay of the curve as a function of age, but this did not give consistent results.

As said above in response to point 7a, we added a new part in New Figure 2 (part A) to clearly explain the Sholl analysis that we performed, and to explain all the terms used in New Figure 2 and New Extended Figure 2-2. We added in the legend of Figure 2A: "Schematic representation of the quantified morphological parameters; the Sholl profiles (B) and morphological parameters (C-H) were obtained by overlaying a template of concentric and equidistant circles onto a two-dimensional images of biocytin-filled neurons with the first circle centered on the somas (radius 0); nodes (blue dots): the points of branching of dendrites; crossing intersections (red dots): the points where the dendritic branches intersect the Sholl circles; critical radius (green): the radius of the circle with the maximum number of crossing intersections; critical value: the number of crossing intersections at critical radius; ending radius (blue): the maximal radius of the circle with crossing intersection(s); Sholl profile is the number of crossing intersections as a function of the radial distance from the cell soma (adapted from Sholl 1953 and Rietveld et al. 2015).

11. In order to understand why whole cell recordings were performed at +10 and -70mV, the authors should mention the reversal potential of their GABAergic and glutamatergic synaptic currents, calculated from their intra and extracellular solutions and illustrated as representative traces with the relevant pharmacological conditions.

Response: The Nernst equation gave the following results for whole cell recordings. The reversal potential of chloride ions, ECl, is -74 mV and the reversal potential of monovalent cations, Ecations is 0 mV.

We added these values in the Methods page 5 lines 109-110 and 119-121.

12. In Fig.4, the sag appears highest at P7 in the traces but lower in the plots (in which it actually looks like the sag disappears after P7-14). This apparent contradiction should be clarified.

Response: For the sag, the misunderstanding came from the fact that we illustrated sags in response to different current steps amplitudes as said in the legend (from -40 pA at E18 to -100 pA at P7). We have now illustrated sags in response to -40 pA everywhere and -100 pA except for E18 and P0 (due to the high membrane resistance these large amplitude current steps could not be applied).

13. In Fig4, post-hyperpolarization rebound firing is visible at all ages except P0. Please comment if the trace is not representative or if something particular takes place at P0. The potential relevance of rebound firing (e.g. as a neuronal synchronization mechanism) should also be properly addressed in the text (eg discussion). It could by the way be quite interesting to investigate the possible relationships between sag expression and rebound firing.

Response: This was due to the difference of the current step used. We have now shown better examples.

Minor issues 14. On p.10, "isoguvacine increased spike activity to 2.65 of control" is not correct. You may for instance consider 2.65 times that of control or increased by 165% compared to control.

Response: We corrected the text accordingly (line 246).

15. On p.11 " GABAA R mediated PSCs had a depolarizing action (Figure 6) that can summate with that of AMPAR-mediated PSCs.". In the literature, synergistic interactions between GABA and NMDA transmission (rather than AMPA-R) has been emphasized. The authors may mention this relevant element, and clarify whether immature Ach neurons also have NMDA-mediated synaptic glutamatergic transmission.

Response: Sorry for this awkward sentence. We wanted to say that both AMPAR-mediated and GABAAR-mediated synaptic transmissions excite neuronal activity, not that their EPSPs summate. We have corrected the text accordingly page 11 lines 264-267.
