# Supplementary material for: Basal Forebrain Cholinergic Neurons Have Specific Characteristics during the Perinatal Period
Source: eNeuro. 2024 May 24;11(5):ENEURO.0538-23.2024. doi: 10.1523/ENEURO.0538-23.2024 (PMC11137802; doi:10.1523/ENEURO.0538-23.2024)
Supplement: Table 2-1 — Statistical analysis related to Figure 2 and Extended Data Figure 2-2. Summary of statistical tests for Figure 2 C-H and Extended Data Figure 2-2 E-K. 95% C.I. of diff - confidence interval for effect size. Download Table 2-1, DOCX file. [file eneuro-11-ENEURO.0538-23.2024-s005.docx]

**Extended Data Table 2-1**

Statistical analysis related to **Figure 2**

| Groups | n (cells) | N (mice) | Sholl area under curve (A.U.)  Mean ± SEM | Total dendritic length (µm)  Mean ± SEM | Critical value (Nb of crossing intersections)  Mean ± SEM | Number of dendritic nodes  Mean ± SEM | Ending radius (µm)  Mean ± SEM | Critical radius (µm)  Mean ± SEM | Number of dendritic truncs  Mean ± SEM |
| --- | --- | --- | --- | --- | --- | --- | --- | --- | --- |
|  |  |  | **Panel C** | **Panel D** | **Panel E** | **Panel F** | **Panel G** | **Panel H** |  |
| **E18** | 15 | 4 | 687± 68 | 1012± 109 | 11.02± 0.78 | 28.47± 3.20 | 145.6± 14.5 | 29.77± 3.99 | 4.67± 0.71 |
| **P0/1** | 15 | 7 | 977± 129 | 1354± 172 | 9.44± 0.85 | 25.60± 4.24 | 221.7± 17.7 | 37.53± 5.80 | 3.93± 0.33 |
| **P2/3** | 14 | 6 | 1548±179 | 2144± 247 | 11.06± 0.89 | 41.71±6.21 | 284.5± 31.4 | 58.84±10.53 | 4.21± 0.21 |
| **P4/5** | 17 | 6 | 1136±95 | 1533± 125 | 7.73± 0.98 | 19.88± 3.27 | 314.0± 24.9 | 84.68±14.93 | 3.29± 0.22 |
| **P6/7** | 17 | 6 | 1026±137 | 1448± 203 | 7.15± 0.56 | 17.18± 2.95 | 268.0± 26.9 | 65.66± 6.85 | 3.12± 0.28 |
| **P8/9** | 16 | 5 | 1009±130 | 1344± 173 | 6.25± 0.41 | 11.44± 1.90 | 323.9± 36.2 | 67.54±11.59 | 3.69± 0.31 |
| **P10/11** | 14 | 6 | 1567±172 | 2198± 251 | 7.77± 0.61 | 14.50± 1.73 | 358.3± 27.9 | 107.9±14.6 | 3.71± 0.27 |
| **P12/13** | 14 | 4 | 1261±124 | 1827±178 | 7.76± 0.79 | 12.21± 2.63 | 300.1± 26.7 | 88.17±12.53 | 3.36± 0.20 |
| **P14/15** | 16 | 7 | 1720±184 | 2414± 251 | 8.44± 0.58 | 13.31± 1.71 | 375.7± 29.3 | 92.08±11.53 | 3.56± 0.33 |

| **Panel C**  Sholl area under curve (A.U.)  groups | Data structure | Test | Adjusted P value* | Power analysis | |
| --- | --- | --- | --- | --- | --- |
| **E18 vs P2/3** | Normal distribution | Two-tailed two-samples t-test | 3*e^-4^ |  |  |
|  |  |  |  | Effect size | 1.77 |
|  |  |  |  | 95% C.I. of diff | 1.010 to 2.31 |
| **P2/3 vs P8/9** | Normal distribution |  | 0.01961 |  |  |
|  |  |  |  | Effect size | -0.881 |
|  |  |  |  | 95% C.I. of diff | -1.61, -0.084 |
| **P8/9 vs P14/15** | Normal distribution |  | 0.0072 |  |  |
|  |  |  |  | Effect size | 1.09 |
|  |  |  |  | 95% C.I. of diff | 0.349, 1.78 |

| **Panel D**  Total dendritic length (µm) groups | Data structure | Test | Adjusted P value* | Power analysis | |
| --- | --- | --- | --- | --- | --- |
| **E18 vs P2/3** | Normal distribution | Two-tailed two-samples t-test | 3*e^-4^ |  |  |
|  |  |  |  | Effect size | 1.65 |
|  |  |  |  | 95% C.I. of diff | 0.909 to 2.133 |
| **P2/3 vs P8/9** | Normal distribution |  | 0.0116 |  |  |
|  |  |  |  | Effect size | -0.962 |
|  |  |  |  | 95% C.I. of diff | -1.65, -0.174 |
| **P8/9 vs P14/15** | Normal distribution |  | 0.00288 |  |  |
|  |  |  |  | Effect size | 1.21 |
|  |  |  |  | 95% C.I. of diff | 0.464, 1.91 |

| **Panel E**  Critical value  groups | Data structure | Test | Adjusted P value | Power analysis | |
| --- | --- | --- | --- | --- | --- |
| **E18 vs P2/3** | Normal distribution | Two-tailed two-samples t-test | 0.97243 | Power | 0.05013 |
|  |  |  |  | Effect size | 0.0126 |
|  |  |  |  | 95% C.I. of diff | -0.712, 0.766 |
| **P2/3 vs P8/9** |  |  | 6*e^-4^ |  |  |
|  |  |  |  | Effect size | -1.83 |
|  |  |  |  | 95% C.I. of diff | -2.77, -0.927 |
| **P8/9 vs P14/15** | Normal distribution |  | 0.0088 |  |  |
|  |  |  |  | Effect size | 1.06 |
|  |  |  |  | 95% C.I. of diff | 0.284, 1.75 |

| **Panel F**  Number of dendritic nodes  groups | Data structure | Test | Adjusted P value* | Power analysis | |
| --- | --- | --- | --- | --- | --- |
| **E18 vs P2/3** | Normal distribution | Two-tailed two-samples t-test | 0.064 | Power | 0.46272 |
|  |  |  |  | Effect size | 0.7 |
|  |  |  |  | 95% C.I. of diff | -0.0123, 1.38 |
| **P2/3 vs P8/9** |  |  | 1*e^-4^ |  |  |
|  |  |  |  | Effect size | -1.76 |
|  |  |  |  | 95% C.I. of diff | -2.32, -1.14 |
| **P8/9 vs P14/15** | Normal distribution |  | 0.47 | Power | 0.11 |
|  |  |  |  | Effect size | 0.253 |
|  |  |  |  | 95% C.I. of diff | -0.479, 0.954 |

| **Panel G**  Ending radius (µm)  groups | Data structure | Test | Adjusted P value* | Power analysis | |
| --- | --- | --- | --- | --- | --- |
| **E18 vs P4/5** | Normal distribution | Two-tailed two-samples t-test | 8*e^-6^ |  |  |
|  |  |  |  | Effect size | 2.06 |
|  |  |  |  | 95% C.I. of diff | 1.115 to 2.71 |
| **P4/5 vs P14/15** | Normal distribution |  | 0.12 | Effect size | 0.345 |
|  |  |  |  | Cohen’s | 0.579 |
|  |  |  |  | 95% C.I. of diff | -0.141 to 1.211 |

| **Panel H**  Critical radius (µm)  groups | Data structure | Test | Adjusted P value* | Power analysis | |
| --- | --- | --- | --- | --- | --- |
| **E18 vs P4/5** | Normal distribution | Two-tailed two-samples t-test | 0.004 |  |  |
|  |  |  |  | Effect size | 1.228 |
|  |  |  |  | 95% C.I. of diff | 0.495 to 1.657 |
| **P4/5 vs P14/15** | Normal distribution |  | 0.7 | Power | 0.066 |
|  |  |  |  | Effect size | 0.140 |
|  |  |  |  | 95% C.I. of diff | -0.60 to 0.799 |

| Number of primary dendrites  groups | Data structure | Test | Adjusted P value* | Power analysis | |
| --- | --- | --- | --- | --- | --- |
| **E18 vs P6/7** | Normal distribution | Two-tailed two-samples t-test | 0.088 | Power | 0.532 |
|  |  |  |  | Effect size | 0.771 |
|  |  |  |  | 95% C.I. of diff | -1.149 to -0.086 |
| **P6/7 vs P14/15** | Normal distribution |  | 0.31 | Power | 0.169 |
|  |  |  |  | Effect size | 0.369 |
|  |  |  |  | 95% C.I. of diff | -0.383 to 1.022 |

*- Holm-Bonferroni Sequential Correction: An EXCEL Calculator" © Justin Gaetano, 2013

Statistical analysis related to **Extended data Figure 2-2**

| Groups | n (cells) | N (mice) | Sholl area under curve (A.U.)  Mean ± SEM | Total dendritic length (µm)  Mean ± SEM | Critical value (Nb of crossing intersections)  Mean ± SEM | Number of dendritic nodes  Mean ± SEM | Ending radius (µm)  Mean ± SEM | Critical radius (µm)  Mean ± SEM | Number of dendritic truncs  Mean ± SEM |
| --- | --- | --- | --- | --- | --- | --- | --- | --- | --- |
|  |  |  | **Panel E** | **Panel F** | **Panel G** | **Panel H** | **Panel I** | **Panel J** | **Panel K** |
| **P0/1 EGFP-** | 15 | 4 | 1036± 70 | 1519± 98 | 8.02± 0.55 | 27.00± 3.23 | 260.2±20.2 | 56.22±10.17 | 4.00± 0.38 |
| **P4/5 EGFP-** | 17 | 3 | 787± 99 | 1112± 147 | 7.01± 0.55 | 17.59± 3.53 | 213.0±20.7 | 46.48± 4.94 | 3.67± 0.19 |
| **P10/11 EGFP-** | 13 | 3 | 936± 103 | 1343± 145 | 6.34± 0.51 | 11.08± 0.93 | 264.0±14.1 | 80.53±10.33 | 3.38± 0.18 |
| **P14/15 EGFP-** | 12 | 6 | 1466± 221 | 2105± 293 | 7.64± 0.98 | 21.92± 4.2 | 355.9±27.6 | 99.79±14.70 | 3.50± 0.42 |

| **Panel E**  Sholl area under curve (A.U.)  Groups | Data structure | Test | P value | Power | |
| --- | --- | --- | --- | --- | --- |
| **EGFP^+^ vs EGFP^-^  P0/1** | Normal distribution | Welch’s t-test | p=0.69 | Power | < 0.1 |
|  |  |  |  | Effect size | 0.148 |
|  |  |  |  | 95% C.I. of diff | -0.615, 0.973 |
| **EGFP^+^ vs EGFP^-^ P4/5** | Non Normal distribution | Mann-Whitney test | p=0.009 |  |  |
|  |  |  |  | Effect size | -0.875 |
|  |  |  |  | 95% C.I. of diff | -1.71, -0.0125 |
| **EGFP^+^ vs EGFP^-^ P10/11** | Non Normal distribution | Mann-Whitney test | p=0.009 | Power | 0.8455 |
|  |  |  |  | Effect size | -1.15 |
|  |  |  |  | 95% C.I. of diff | -2.01, -0.262 |
| **EGFP^+^ vs EGFP^-^ P14/15** | Non Normal distribution | Mann-Whitney test | p=0.347 | Power | < 0.1 |
|  |  |  |  | Effect size | -0.329 |
|  |  |  |  | 95% C.I. of diff | -1.12, 0.516 |

| **Panel F**  Total dendritic length (µm)  Groups |  | Test | P value | Power | |
| --- | --- | --- | --- | --- | --- |
| **EGFP^+^ vs EGFP^-^ P0/1** | Normal distribution | Welch’s t-test | p=0.41 | Power | 0.12705 |
|  |  |  |  | Effect size | 0.318 |
|  |  |  |  | 95% C.I. of diff | -0.489, 1.14 |
| **EGFP^+^ vs EGFP^-^ P4/5** | Non Normal distribution | Mann-Whitney test | p=0.008 |  | 0.57 |
|  |  |  |  | Effect size | -0.748 |
|  |  |  |  | 95% C.I. of diff | -1.61, 0.0976 |
| **EGFP^+^ vs EGFP^-^ P10/11** | Non Normal distribution | Mann-Whitney test | p=0.013 |  |  |
|  |  |  |  | Effect size | -1.079 |
|  |  |  |  | 95% C.I. of diff | -1.91, -0.241 |
| **EGFP^+^ vs EGFP^-^ P14/15** | Non Normal distribution | Mann-Whitney test | p=0.35 | Power | < 0.1 |
|  |  |  |  | Effect size | -0.215 |
|  |  |  |  | 95% C.I. of diff | -0.923, 0.549 |

| **Panel G**  Critical value  Groups | Data structure | Test | P value | Power | |
| --- | --- | --- | --- | --- | --- |
| **EGFP^+^ vs EGFP^-^ P0/1** | Non Normal distribution | Mann-Whitney test | p=0.054 | Power | 0.2755 |
|  |  |  |  | Effect size | -0.509 |
|  |  |  |  | 95% C.I. of diff | -1.24, 0.365 |
| **EGFP^+^ vs EGFP^-^ P4/5** | Non Normal distribution | Mann-Whitney test | p=0.94 | Power | < 0.1 |
|  |  |  |  | Effect size | -0.22 |
|  |  |  |  | 95% C.I. of diff | -0.878, 0.492 |
| **EGFP^+^ vs EGFP^-^ P10/11** | Non Normal distribution | Mann-Whitney test | p=0.078 | Power | 0.418 |
|  |  |  |  | Effect size | -0.671 |
|  |  |  |  | 95% C.I. of diff | -1.34, 0.242 |
| **EGFP^+^ vs EGFP^-^ P14/15** | Normal distribution | Two-sample 2-tailed t - test | p=0.46 | Power | 0.11 |
|  |  |  |  | Effect size | -0.275 |
|  |  |  |  | 95% C.I. of diff | -1.16, 0.557 |

| **Panel H**  Number of dendritic nodes  Groups | Data structure | Test | P value | Power | |
| --- | --- | --- | --- | --- | --- |
| **EGFP^+^ vs EGFP^-^ P0/1** | Non Normal distribution | Mann-Whitney test | p=0.53 | Power | < 0.1 |
|  |  |  |  | Effect size | 0.0959 |
|  |  |  |  | 95% C.I. of diff | -0.693 to 0.882 |
| **EGFP^+^ vs EGFP^-^ P4/5** | Non Normal distribution | Mann-Whitney test | p=0.36 | Power | < 0.1 |
|  |  |  |  | Effect size | -0.164 |
|  |  |  |  | 95% C.I. of diff | -0.848 to 0.538 |
| **EGFP^+^ vs EGFP^-^ P10/11** | Normal distribution | Welch’s t-test | p=0.097 | Power | 0.375 |
|  |  |  |  | Effect size | 0.637 |
|  |  |  |  | 95% C.I. of diff | -0.127 to 1.27 |
| **EGFP^+^ vs EGFP^-^ P14/15** | Non Normal distribution | Mann-Whitney test | p=0.16 | Power | 0.532 |
|  |  |  |  | Effect size | 0.771 |
|  |  |  |  | 95% C.I. of diff | -0.0784 to 1.63 |

| **Panel I**  Ending radius (µm)  Groups | Data structure | Test | P value | Power | |
| --- | --- | --- | --- | --- | --- |
| **EGFP^+^ vs EGFP^-^ P0/1** | Normal distribution | Two-sample 2-tailed t - test | p=0.16 | Power | 0.28421 |
|  |  |  |  | Effect size | 0.525 |
|  |  |  |  | 95% C.I. of diff | -0.238, 1.32 |
| **EGFP^+^ vs EGFP^-^ P4/5** | Normal distribution | Two-sample 2-tailed t - test | p=0.0038 |  |  |
|  |  |  |  | Effect size | -1.07 |
|  |  |  |  | 95% C.I. of diff | -1.83, -0.281 |
| **EGFP^+^ vs EGFP^-^ P10/11** | Normal distribution | Welch’s t-test | p=0.007 |  |  |
|  |  |  |  | Effect size | -1.1 |
|  |  |  |  | 95% C.I. of diff | -1.81, -0.335 |
| **EGFP^+^ vs EGFP^-^ P14/15** | Normal distribution | Two-sample 2-tailed t - test | p=0.636 | Power | 0.07471 |
|  |  |  |  | Effect size | -0.177 |
|  |  |  |  | 95% C.I. of diff | -0.895, 0.56 |

| **Panel J**  Critical radius (µm)  Groups | Data structure | Test | P value | Power | |
| --- | --- | --- | --- | --- | --- |
| **EGFP^+^ vs EGFP^-^ P0/1** | Non Normal distribution | Mann-Whitney test | p=0.15 | Power | 0.342 |
|  |  |  |  | Effect size | 0.583 |
|  |  |  |  | 95% C.I. of diff | -0.213, 1.16 |
| **EGFP^+^ vs EGFP^-^ P4/5** | Non Normal distribution | Mann-Whitney test | p=0.022 |  |  |
|  |  |  |  | Effect size | -0.834 |
|  |  |  |  | 95% C.I. of diff | -1.31, -0.164 |
| **EGFP^+^ vs EGFP^-^ P10/11** | Normal distribution | Two-sample 2-tailed t - test | p=0.14 | Power | 0.30451 |
|  |  |  |  | Effect size | -0.562 |
|  |  |  |  | 95% C.I. of diff | -1.3, 0.219 |
| **EGFP^+^ vs EGFP^-^ P14/15** | Non Normal distribution | Mann-Whitney test | p=0.66 | Power | < 0.1 |
|  |  |  |  | Effect size | 0.155 |
|  |  |  |  | 95% C.I. of diff | -0.639, 0.958 |

| **Panel K**  Number of primary dendrites  Groups | Data structure | Test | P value | Power | |
| --- | --- | --- | --- | --- | --- |
| **EGFP^+^ vs EGFP^-^P0/1** | Non Normal distribution | Mann-Whitney test | p=0.81 | Power | < 0.1 |
|  |  |  |  | Effect size | 0.0485 |
|  |  |  |  | 95% C.I. of diff | -0.757, 0.755 |
| **EGFP^+^ vs EGFP^-^ P4/5** | Non Normal distribution | Mann-Whitney test | p=0.21 | Power | 0.209 |
|  |  |  |  | Effect size | 0.413 |
|  |  |  |  | 95% C.I. of diff | -0.345, 1.13 |
| **EGFP^+^ vs EGFP^-^ P10/11** | Non Normal distribution | Mann-Whitney test | p=0.43 | Power | 0.1615 |
|  |  |  |  | Effect size | -0.377 |
|  |  |  |  | 95% C.I. of diff | -1.07, 0.375 |
| **EGFP^+^ vs EGFP^-^ P14/15** | Non Normal distribution | Mann-Whitney test | p=0.76 | Power | < 0.1 |
|  |  |  |  | Effect size | -0.0442 |
|  |  |  |  | 95% C.I. of diff | -0.781, 0.77 |
