# Supplementary figures and images for: Basal Forebrain Cholinergic Neurons Have Specific Characteristics during the Perinatal Period
Source: eNeuro. 2024 May 24;11(5):ENEURO.0538-23.2024. doi: 10.1523/ENEURO.0538-23.2024 (PMC11137802; doi:10.1523/ENEURO.0538-23.2024)

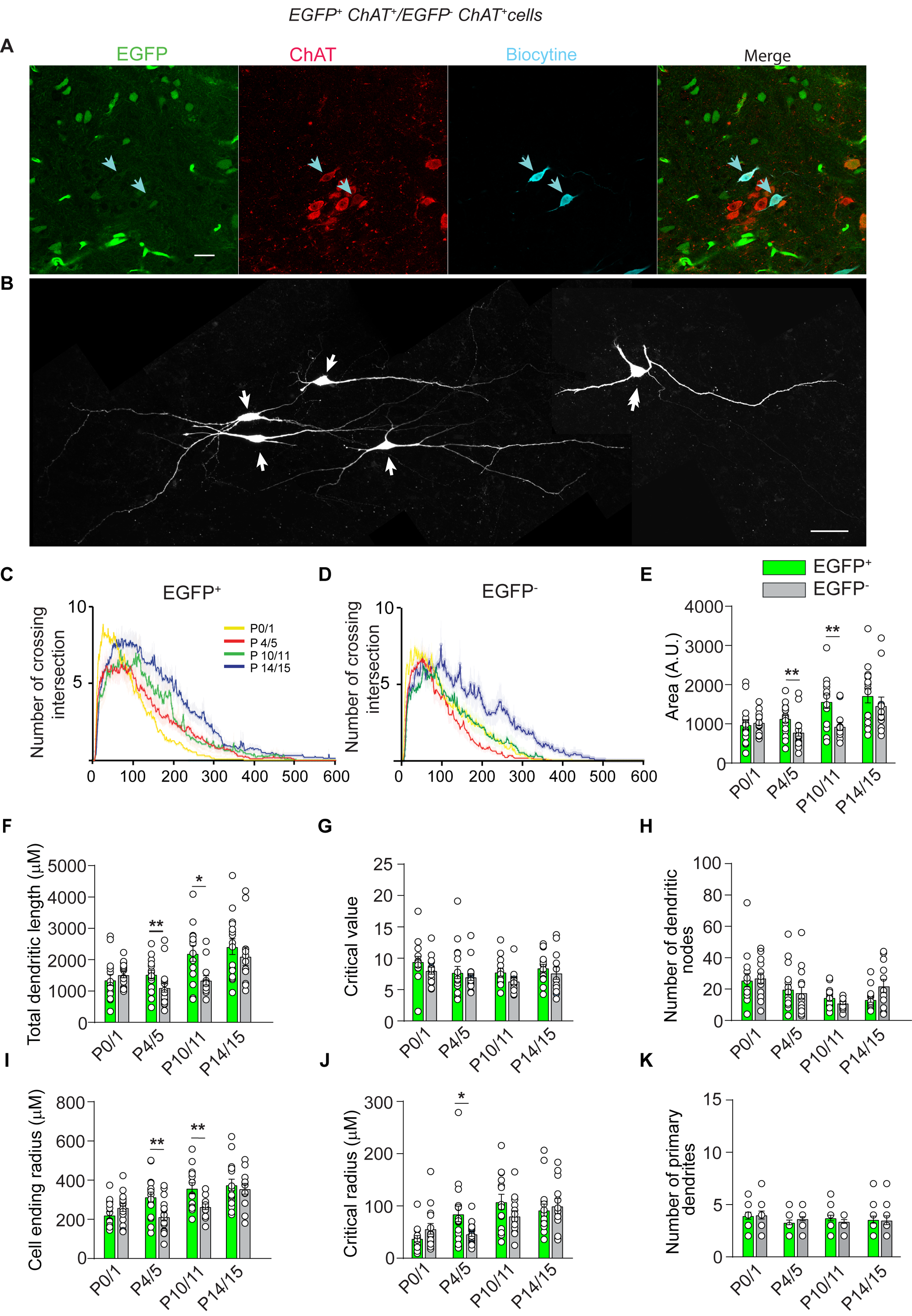

Supplement: Figure 2-2 — Comparison of the dendritic parameters of GABAergic and non-GABAergic cholinergic neurons (P0-15). A, Example of ChAT staining (red) in two EGFP-, biocytin-filled (blue) neurons (arrows) in a slice from a Lhx6-iCre;RCE-EGFP+ mouse. Scale bar: 30 µm. B, Representative biocytin-filled cholinergic/GABAergic (arrows) and cholinergic/non-GABAergic (double arrow) neurons at P5. Scale bar: 50 µm. C, Mean Sholl profiles for cholinergic/GABAergic neurons, and D, Mean Sholl profiles of cholinergic/non-GABAergic neurons at the indicated ages. E-K, Comparison of dendritic parameters (as indicated in ordinates and see Figure 2A) of biocytin-filled GABAergic (green) and non-GABAergic (grey) cholinergic neurons during development (abscissae). Cholinergic/GABAergic: (P0/1: n=15, N=7; P4/5: n= 17, N=6; P10/11: n=14, N=6; P14/15: n=16, N=7). Cholinergic/non-GABAergic:(P0/1: n=15, N=4; P4/5: n= 17, N=3; P10/11: n=13, N=3; P12/13: n=14, N=6; P14/15: n=12, N=6). n=number of cells, N=number of mice. Mean ± SEM, *: P < 0.05; **: P < 0.01; ***P: < 0.001. (Extended Data Table 2-1). Download Figure 2-2, TIF file. [file eneuro-11-ENEURO.0538-23.2024-s002.tif]

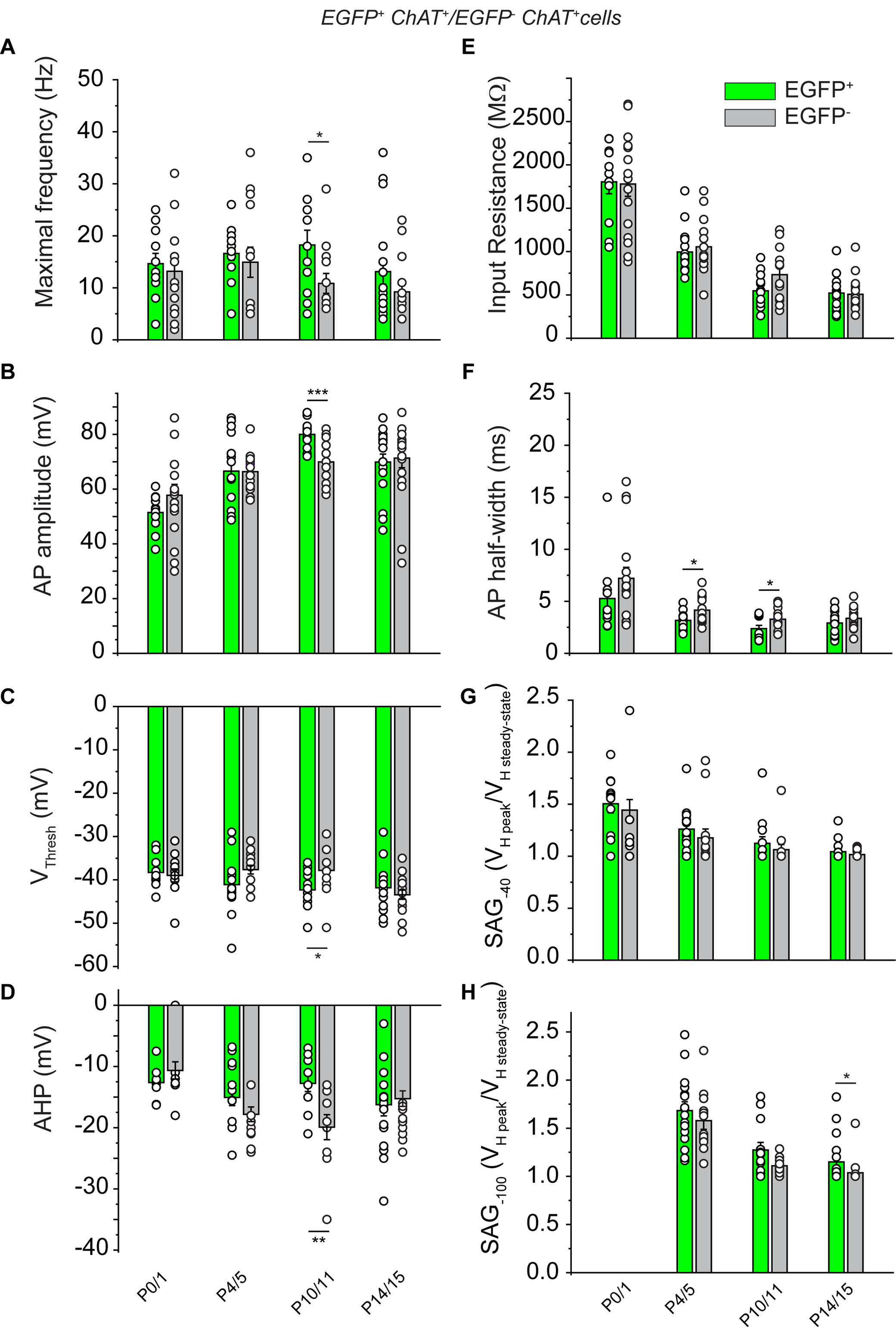

Supplement: Figure 3-2 — Comparison of the intrinsic electrophysiological properties of GABAergic and non-GABAergic cholinergic neurons (P0-P15). A-F, Quantification of the main electrophysiological properties (as indicated in ordinates and see Figure 3A) of GABAergic (green) and non-GABAergic (grey) cholinergic neurons during development. (EGFP+: P0/1: n=11, N=4; P4/5: n= 15, N=7; P6/7: n= 19, N=7; P10/11: n=13, N=4; P14/15: n=17, N=6; EGFP-: P0/1: n=17, N=5; P4/5: n= 14, N=3; P10/11: n=13, N=3; P14/15: n=18, N=5). G, Mean sag amplitude in response to a -40 pA hyperpolarizing pulse (Sag-40), (EGFP+: P0/1: n=11, N=4; P4/5: n= 16, N=8; P10/11: n=13, N=4; P14/15: n=17, N=6; EGFP-: P0/1: n=17, N=5; P4/5: n= 13, N=3; P10/11: n=13, N=3; P14/15: n=18, N=5). H, Mean sag amplitude in response to a -100 pA hyperpolarizing pulse (Sag-100), (EGFP+: P4/5: n= 16, N=8; P10/11: n=13, N=4; P14/15: n=17, N=6; EGFP-: P4/5: n= 13, N=3; P10/11: n=13, N=3; P14/15: n=18, N=5) at the indicated ages. n=number of cells, N=number of mice. Mean ± SEM, *: P < 0.05; **: P < 0.01; ***P: < 0.001 (Extended Data Table 3-1). Download Figure 3-2, TIF file. [file eneuro-11-ENEURO.0538-23.2024-s003.tif]

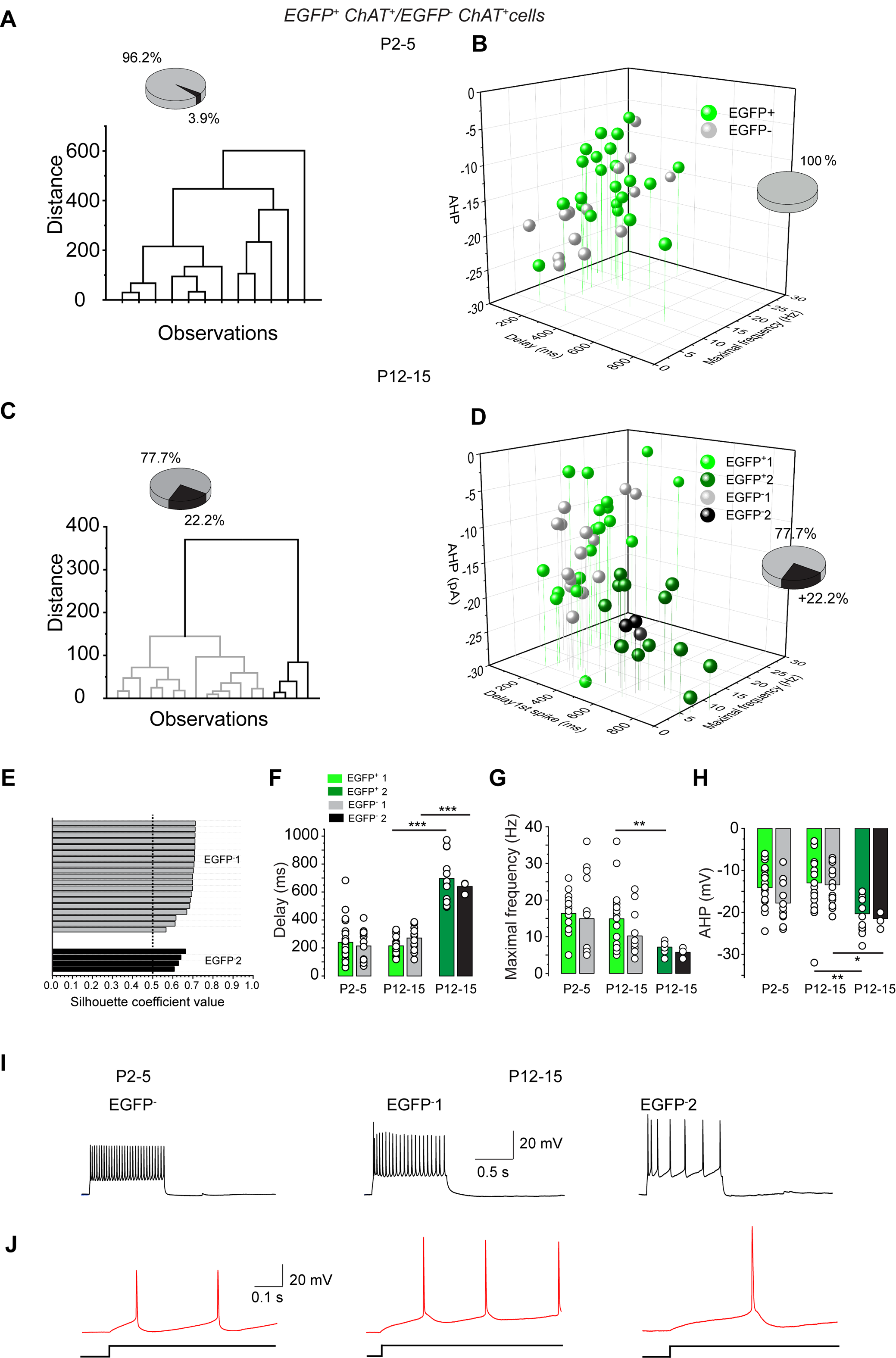

Supplement: Figure 4-2 — Cluster analysis of GABAergic and non-GABAergic cholinergic neurons (P2-5 and P12-15) based on intrinsic electrophysiological properties. A, Hierarchical clustering dendrogram for the intrinsic electrophysiological properties of P2-5 cholinergic/non-GABAergic neurons: delay of the 1st spike in response to a juxta threshold depolarization, AP amplitude, maximal frequency, VThresh, AHP amplitude, half-width of evoked AP, Sag amplitudes in response to -40 pA and -100 pA. B, 3-D plot of the unique cluster of cholinergic/non-GABAergic neurons (EGFP-, grey) obtained from the k-means algorithm; Parameters: AHP amplitude, delay of the 1st spike in response to juxta threshold depolarization and maximal frequency (P2-P5, n=14, N=3). The unique cluster of cholinergic/GABAergic neurons (EGFP+, green, see Fig. 5B) is superimposed. C, Hierarchical clustering dendrogram for the intrinsic electrophysiological properties of P12-15 cholinergic/non-GABAergic neurons: delay of the 1st spike in response to a juxta threshold depolarization, AP amplitude, maximal frequency, VThresh, AHP amplitude, half-width of evoked AP, Sag amplitudes in response to -50 pA and -100 pA (n=28, N=9). D, 3D-plot of the two clusters obtained from the k-means algorithm for cholinergic/non-GABAergic neurons: EGFP-1 (grey) and EGFP-2 (black) (P12-P15, n=14, N= 6 and n=4, N=3, respectively); Parameters: AHP amplitude, delay of the 1st spike in response to juxta threshold depolarization and maximal frequency. The two clusters obtained for P12-15 cholinergic/GABAergic neurons (green and dark green, see Fig. 5D) are superimposed. Insets in A,B and C,D show percentage of cells belonging to each cluster. E, Silhouette coefficient plot for EGFP-1 and EGFP-2 clusters. F-H, Quantification of delay of the 1st spike in response to juxta threshold depolarization, maximal frequency and AHP amplitude of GABAergic (green) and non-GABAergic (grey) cholinergic neurons from dataset at P2-5 and 4 clusters obtained from the k-mean [file eneuro-11-ENEURO.0538-23.2024-s004.tif]
