# Supplementary material for: Basal Forebrain Cholinergic Neurons Have Specific Characteristics during the Perinatal Period
Source: eNeuro. 2024 May 24;11(5):ENEURO.0538-23.2024. doi: 10.1523/ENEURO.0538-23.2024 (PMC11137802; doi:10.1523/ENEURO.0538-23.2024)
Supplement: Table 3-1 — Statistical analysis related to Figure 3 and Extended Data Figure 3-2. Summary of statistical tests for Figure 3 B-I and Extended Data Figure 3-2 A-H. 95% C.I. of diff - confidence interval for effect size. Download Table 3-1, DOCX file. [file eneuro-11-ENEURO.0538-23.2024-s006.docx]

**Extended Data Table 3-1**

Statistical analysis related to **Figure 3.**

| Groups | n (cells) | N (mice) | Maximal frequency (Hz)  Mean ± SEM  **Panel B** | AP amplitude (pA)  Mean ± SEM  **Panel C** | Vthresh (mV)  Mean ± SEM  **Panel D** | AHP (mV)  Mean ± SEM  **Panel E** | Input resistance (MΩ)  Mean ± SEM  **Panel F** | AP half-width (ms)  Mean ± SEM  **Panel G** |
| --- | --- | --- | --- | --- | --- | --- | --- | --- |
| **E18** | 13 | 4 | 9.46±1.99 | 47.08± 2.24 | 33.54±1.281 | -10.46± 1.53 | 2494±255 | 7.70±0.80 |
| **P0/1** | 11 | 4 | 14.64±2.00 | 51.47±2.02 | 38.28±1.044 | -12.64± 0.72 | 1804±137 | 5.27±1.06 |
| **P2/3** | 14 | 6 | 19.57±0.94 | 56.93±2.39 | 39.43±1.09 | -12.85± 1.08 | 1323±110 | 3.65±0.17 |
| **P4/5** | 15 | 7 | 16.6±1.29 | 66.57±3.44 | -41.1±1.62 | -15.06± 1.33 | 994±70 | 3.17±0.20 |
| **P6/7** | 19 | 7 | 16.47±1.76 | 72.47±1.51 | -39.91± 0.89 | -15.7±1.19 | 689±43 | 3.14±0.18 |
| **P8/9** | 12 | 4 | 17.25±1.81 | 69.5±2.37 | -41.42± 1.69 | -15.11± 1.34 | 665±65 | 3.35±0.19 |
| **P10/11** | 13 | 4 | 18.23±2.84 | 80±1.35 | -42.31± 1.15 | -12.77± 1.32 | 548±53 | 2.37±0.32 |
| **P12/13** | 14 | 3 | 11.57±1.50 | 71.57± 2.21 | -41.64± 1.04 | -15.46± 1.75 | 509±23 | 3.60±0.30 |
| **P14/15** | 17 | 6 | 13.12±2.34 | 69.88± 2.93 | -41.82± 1.33 | -16.24±1.83 | 522±47 | 2.92±0.27 |

| Groups | n (cells) | N (mice) | Sag amplitude at -40  Mean ± SEM  **Panel H** | n (cells) | N (mice) | Sag amplitude at -100  Mean ± SEM  **Panel I** |
| --- | --- | --- | --- | --- | --- | --- |
| **E18** | 12 | 4 | 1.39±0.09 |  |  |  |
| **P0/1** | 11 | 4 | 1.51±0.09 |  |  |  |
| **P2/3** | 12 | 6 | 1.30±0.08 | 8 | 6 | 1.56±0.13 |
| **P4/5** | 16 | 8 | 1.26±0.05 | 16 | 8 | 1.68±0.09 |
| **P6/7** | 18 | 7 | 1.13±0.05 | 15 | 7 | 1.50±0.10 |
| **P8/9** | 13 | 5 | 1.14±0.06 | 13 | 5 | 1.19±0.06 |
| **P10/11** | 13 | 4 | 1.12±0.06 | 13 | 4 | 1.27±0.08 |
| **P12/13** | 14 | 3 | 1.07±0.03 | 13 | 3 | 1.13±0.07 |
| **P14/15** | 17 | 6 | 1.04±0.02 | 17 | 6 | 1.15±0.06 |

| **Panel B**  Maximal frequency (Hz)  groups | Data structure | Test | Adjusted P value* | Power | |
| --- | --- | --- | --- | --- | --- |
| **E18 vs P2/3** | Normal distribution | Two-tailed two-samples t-test | 2*e^-4^ |  |  |
|  |  |  |  | Effect size | 1.759 |
|  |  |  |  | 95% C.I. of diff | 0.236 to 3.643 |
| **P2/3 vs P14/15** | Non Normal distribution | Mann-Whitney Test | 0.00378 |  | 0.6 |
|  |  |  |  | Effect size | -0.815 |
|  |  |  |  | 95% C.I. of diff | -1.942 to 0.069 |

| **Panel C**  AP amplitude (pA)  groups | Data structure | Test | Adjusted P value* | Power | |
| --- | --- | --- | --- | --- | --- |
| **E18 vs P6/7** | Normal distribution | Two-tailed two-samples t-test | 2*e^-10^ |  |  |
|  |  |  |  | Effect size | 3.426 |
|  |  |  |  | 95% C.I. of diff | 2.350 to 4.409 |
| **P6/7 vs P14/15** | Normal distribution |  | 0.42 | Power | 0.12301 |
|  |  |  |  | Effect size | -0.263 |
|  |  |  |  | 95% C.I. of diff | -0.938 to 0.434 |

| **Panel D**  V_thresh_ (mV)  groups | Data structure | Test | Adjusted P value* | Power | |
| --- | --- | --- | --- | --- | --- |
| **E18 vs P4/5** | Normal distribution | Two-tailed two-samples t-test | 0.002 |  |  |
|  |  |  |  | Effect size | -1.313 |
|  |  |  |  | 95% C.I. of diff | -2.09 to -0.423 |
| **P4/5 vs P14/15** | Normal distribution |  | 0.73 | Power | 0.063 |
|  |  |  |  | Effect size | -0.1200 |
|  |  |  |  | 95% C.I. of diff | -0.828 to 0.588 |

| **Panel E**  AHP (mV)  groups | Data structure | Test | Adjusted P value* | Power | |
| --- | --- | --- | --- | --- | --- |
| **E18 vs P6/7** | Normal distribution | Two-tailed two-samples t-test | 0.02 |  |  |
|  |  |  |  | Effect size | -0.958 |
|  |  |  |  | 95% C.I. of diff | -1.622 to -0.231 |
| **P6/7 vs P14/15** | Non Normal distribution | Mann-Whitney Test | 0.51519 | Power | < 0.1 |
|  |  |  |  | Effect size | -0.0826 |
|  |  |  |  | 95% C.I. of diff | -0.769 to 0.596 |

| **Panel F**  Input resistance (MΩ)  groups | Data structure | Test | Adjusted P value* | Power | |
| --- | --- | --- | --- | --- | --- |
| **E18 vs P8/9** | Normal distribution | Two-tailed two-samples t-test | 2*e^-6^ |  |  |
|  |  |  |  | Effect size | -2.587 |
|  |  |  |  | 95% C.I. of diff | -3.555 to -1.736 |
| **P8/9 vs P14/15** | Normal distribution |  | 0.079 | Power | 0.422 |
|  |  |  |  | Effect size | -0.670 |
|  |  |  |  | 95% C.I. of diff | -1.4249 to 0.115 |

| **Panel G**  AP half-width (ms)  groups | Data structure | Test | Adjusted P value* | Power | |
| --- | --- | --- | --- | --- | --- |
| **E18 vs P4/5** | Normal distribution | Two-tailed two-samples t-test | 8*e^-6^ |  |  |
|  |  |  |  | Effect size | -2.1557 |
|  |  |  |  | 95% C.I. of diff | -3.212 to -1.279 |
| **P4/5 vs P14/15** | Normal distribution |  | 0.47 | Power | 0.110 |
|  |  |  |  | Effect size | -0.255 |
|  |  |  |  | 95% C.I. of diff | -0.958 to 0.433 |

| **Panel H**  Mean amplitude of SAG (current injection) at -40 pA Groups | Data structure | Test | Adjusted P value* | Power | |
| --- | --- | --- | --- | --- | --- |
| **E18 vs P6/7** | Non Normal distribution | Mann-Whitney Test | 0.0257 |  |  |
|  |  |  |  | Effect size | -1.012 |
|  |  |  |  | 95% C.I. of diff | -1.735 to -0.21 |
| **P6/7 vs P14/15** | Non Normal distribution |  | 0.25666 | Power | 0.342 |
|  |  |  |  | Effect size | -0.539 |
|  |  |  |  | 95% C.I. of diff | -1.076 to 0.096 |

| **Panel I**  Mean amplitude of SAG (current injection) at -100 pA Groups | Data structure | Test | Adjusted P value* | Power | |
| --- | --- | --- | --- | --- | --- |
| **P2/3 vs P8/9** | Non Normal distribution | Mann-Whitney Test | 0.013 |  |  |
|  |  |  |  | Effect size | -1.341 |
|  |  |  |  | 95% C.I. of diff | -2.173 to -0.449 |
| **P8/9 vs P14/15** | Non Normal distribution |  | 0.2644 | Power | < 0.1 |
|  |  |  |  | Effect size | -0.157 |
|  |  |  |  | 95% C.I. of diff | -0.961 to 0.544 |

*- Holm-Bonferroni Sequential Correction: An EXCEL Calculator" © Justin Gaetano, 2013

Statistical analysis related to **Extended data Figure 3-2**

| Groups | n (cells) | N (mice) | Maximal frequency (Hz)  Mean ± SEM  **Panel A** | AP amplitude (pA)  Mean ± SEM  **Panel B** | Vthresh (mV)  Mean ± SEM  **Panel C** | AHP (mV)  Mean ± SEM  **Panel D** | Input resistance (MΩ)  Mean ± SEM  **Panel E** | AP half-width (ms)  Mean ± SEM  **Panel F** |
| --- | --- | --- | --- | --- | --- | --- | --- | --- |
| **P0/1 EGFP-** | 17 | 5 | 13.18±2.04 | 57.76±3.98 | -38.98±1.49 | -10.64±1.40 | 1780±14 | 7.21±1.06 |
| **P4/5 EGFP-** | 14 | 3 | 14.93±2.89 | 66.36±1.86 | -37.64±1.00 | -17.81±1.19 | 1055±86 | 4.15±0.36 |
| **P10/11 EGFP-** | 13 | 3 | 10.85±1.93 | 69.92±2.32 | -37.82±1.49 | -19.92±2.043 | 733±85 | 3.27±0.28 |
| **P14/15 EGFP-** | 18 | 5 | 9.22±1.26 | 71.33±3.53 | -43.43±1.07 | -15.25±1.27 | 507±43 | 3.36±0.23 |

| Groups | n (cells) | N (mice) | Sag amplitude at -40  Mean ± SEM  **Panel G** | n (cells) | N (mice) | Sag amplitude at -100  Mean ± SEM  **Panel H** |
| --- | --- | --- | --- | --- | --- | --- |
| **P0/1 EGFP-** | 17 | 5 | 1.44±0.10 | - | - | - |
| **P4/5 EGFP-** | 13 | 3 | 1.18±0.09 | 13 | 3 | 1.58±0.14 |
| **P10/11 EGFP-** | 13 | 3 | 1.06±0.05 | 12 | 3 | 1.11±0.02 |
| **P14/15 EGFP-** | 18 | 5 | 1.02±0.01 | 18 | 5 | 1.04±0.03 |

| Maximal frequency (Hz)  **Panel A** | Data structure | test | P value | Power | |
| --- | --- | --- | --- | --- | --- |
| **EGFP^+^ vs EGFP^-^  P0/1** | Normal distribution | Two-sample 2-tailed t - test | 0.63 | Power | 0.07544 |
|  |  |  |  | Effect size | -0.182 |
|  |  |  |  | 95% C.I. of diff | -0.948 to 0.549 |
| **EGFP^+^ vs EGFP^-^ P4/5** | Normal distribution | Two-sample 2-tailed t - test | 0.59 | Power | 0.08161 |
|  |  |  |  | Effect size | -0.195 |
|  |  |  |  | 95% C.I. of diff | -1.05 to 0.583 |
| **EGFP^+^ vs EGFP^-^ P10/11** | Normal distribution | Two-sample 2-tailed t - test | 0.042 |  | 0.548 |
|  |  |  |  | Effect size | -0.817 |
|  |  |  |  | 95% C.I. of diff | -1.65 to 0.0285 |
| **EGFP^+^ vs EGFP^-^ P14/15** | Non Normal distribution | Mann-Whitney test | 0.20 | Power | 0.304 |
|  |  |  |  | Effect size | -0.483 |
|  |  |  |  | 95% C.I. of diff | -1.08 to 0.167 |

| AP amplitude (pA)  **Panel B** | Data structure | test | P value | Power | |
| --- | --- | --- | --- | --- | --- |
| **EGFP^+^ vs EGFP^-^  P0/1** | Normal distribution | Two-sample 2-tailed t - test | 0.24 | Power | 0.21178 |
|  |  |  |  | Effect size | -0.128 |
|  |  |  |  | 95% C.I. of diff | -0.241 to 1.11 |
| **EGFP^+^ vs EGFP^-^ P4/5** | Normal distribution | Two-sample 2-tailed t - test | 0.96 | Power | 0.0503 |
|  |  |  |  | Effect size | 0.642 |
|  |  |  |  | 95% C.I. of diff | -0.757 to 0.747 |
| **EGFP^+^ vs EGFP^-^ P10/11** | Normal distribution | Two-sample 2-tailed t - test | 1*E^-3^ |  |  |
|  |  |  |  | Effect size | 0.908 |
|  |  |  |  | 95% C.I. of diff | -2.292 to -0.638 |
| **EGFP^+^ vs EGFP^-^ P14/15** | Normal distribution | Two-sample 2-tailed t - test | 0.76 | Power | 0.06073 |
|  |  |  |  | Effect size | -0.314 |
|  |  |  |  | 95% C.I. of diff | -0.582 0.794 |

| Vthresh (mV)  **Panel C** | Data structure | test | P value | Power | |
| --- | --- | --- | --- | --- | --- |
| **EGFP^+^ vs EGFP^-^ P0/1** | Normal distribution | Two-sample 2-tailed t - test | 0.74 | Power | 0.06254 |
|  |  |  |  | Effect size | -0.128 |
|  |  |  |  | 95% C.I. of diff | -0.774 to 0.569 |
| **EGFP^+^ vs EGFP^-^ P4/5** | Normal distribution | Two-sample 2-tailed t - test | 0.09 | Power | 0.403 |
|  |  |  |  | Effect size | 0.642 |
|  |  |  |  | 95% C.I. of diff | -0.146 to 1.3 |
| **EGFP^+^ vs EGFP^-^ P10/11** | Normal distribution | Two-sample 2-tailed t - test | 0.025 |  | 0.631 |
|  |  |  |  | Effect size | 0.938 |
|  |  |  |  | 95% C.I. of diff | -0.141 to 1.787 |
| **EGFP^+^ vs EGFP^-^ P14/15** | Normal distribution | Two-sample 2-tailed t - test | 0.35 | Power | 0.15181 |
|  |  |  |  | Effect size | -0.314 |
|  |  |  |  | 95% C.I. of diff | -0.975 to 0.359 |

| AHP (mV)  **Panel D** | Data structure | test | P value | Power | |
| --- | --- | --- | --- | --- | --- |
| **EGFP^+^ vs EGFP^-^ P0/1** | Normal distribution | Two-sample 2-tailed t - test | 0.29 | Power | 0.18052 |
|  |  |  |  | Effect size | 0.406 |
|  |  |  |  | 95% C.I. of diff | -0.253 to 1.08 |
| **EGFP^+^ vs EGFP^-^ P4/5** | Normal distribution | Two-sample 2-tailed t - test | 0.14 | Power | 0.31572 |
|  |  |  |  | Effect size | -0.554 |
|  |  |  |  | 95% C.I. of diff | -1.303 to 0.242 |
| **EGFP^+^ vs EGFP^-^ P10/11** | Normal distribution | Two-sample 2-tailed t - test | 0.006 |  |  |
|  |  |  |  | Effect size | -1.196 |
|  |  |  |  | 95% C.I. of diff | -1.94 to -0.384 |
| **EGFP^+^ vs EGFP^-^ P14/15** | Non Normal distribution | Two-sample 2-tailed t - test | 0.72588 | Power | < 0.1 |
|  |  |  |  | Effect size | 0.149 |
|  |  |  |  | 95% C.I. of diff | -0.528 to 0.819 |

| Input resistance (MΩ)  **Panel E** | Data structure | test | P value | Power | |
| --- | --- | --- | --- | --- | --- |
| **EGFP^+^ vs EGFP^-^ P0/1** | Normal distribution | Two-sample 2-tailed t - test | 0.91 | Power | 0.05139 |
|  |  |  |  | Effect size | -0.0429 |
|  |  |  |  | 95% C.I. of diff | -0.766 to 0.685 |
| **EGFP^+^ vs EGFP^-^ P4/5** | Normal distribution | Two-sample 2-tailed t - test | 0.58 | Power | 0.0833 |
|  |  |  |  | Effect size | 0.200 |
|  |  |  |  | 95% C.I. of diff | -0.572 to 0.972 |
| **EGFP^+^ vs EGFP^-^ P10/11** | Normal distribution | Two-sample 2-tailed t - test | 0.078 | Power | 0.424 |
|  |  |  |  | Effect size | 0.722 |
|  |  |  |  | 95% C.I. of diff | -0.0834 to 1.48 |
| **EGFP^+^ vs EGFP^-^ P14/15** | Normal distribution | Two-sample 2-tailed t - test | 0.82 | Power | 0.05593 |
|  |  |  |  | Effect size | -0.0772 |
|  |  |  |  | 95% C.I. of diff | -0.734 to 0.621 |

| AP half-width (ms)  **Panel F** | Data structure | test | P value | Power | |
| --- | --- | --- | --- | --- | --- |
| **EGFP^+^ vs EGFP^-^ P0/1** | Normal distribution | Two-sample 2-tailed t - test | 0.23 | Power | 0.22074 |
|  |  |  |  | Effect size | 0.463 |
|  |  |  |  | 95% C.I. of diff | -0.582 to 1.04 |
| **EGFP^+^ vs EGFP^-^ P4/5** | Normal distribution | Two-sample 2-tailed t - test | 0.02 |  |  |
|  |  |  |  | Effect size | 0.884 |
|  |  |  |  | 95% C.I. of diff | 0.178 to 1.56 |
| **EGFP^+^ vs EGFP^-^ P10/11** | Normal distribution | Two-sample 2-tailed t - test | 0.04 |  |  |
|  |  |  |  | Effect size | 0.832 |
|  |  |  |  | 95% C.I. of diff | 0.00765 to 1.71 |
| **EGFP^+^ vs EGFP^-^ P14/15** | Normal distribution | Two-sample 2-tailed t - test | 0.22 | Power | 0.216 |
|  |  |  |  | Effect size | 0.417 |
|  |  |  |  | 95% C.I. of diff | -0.28 to 1.095 |

| Sag amplitude at -40  **Panel G** | Data structure | test | P value | Power | |
| --- | --- | --- | --- | --- | --- |
| **EGFP^+^ vs EGFP^-^ P0/1** | Normal distribution | Two-sample 2-tailed t - test | 0.67 | Power | 0.06935 |
|  |  |  |  | Effect size | -0.159 |
|  |  |  |  | 95% C.I. of diff | -0.96 to 0.596 |
| **EGFP^+^ vs EGFP^-^ P4/5** | Normal distribution | Two-sample 2-tailed t - test | 0.39 | Power | 0.13389 |
|  |  |  |  | Effect size | -0.316 |
|  |  |  |  | 95% C.I. of diff | -1.29 to 0.571 |
| **EGFP^+^ vs EGFP^-^ P10/11** | Non Normal distribution | Mann-Whitney test | 0.23 | Power | < 0.1 |
|  |  |  |  | Effect size | -0.297 |
|  |  |  |  | 95% C.I. of diff | -0.943 to 0.587 |
| **EGFP^+^ vs EGFP^-^ P14/15** | Non Normal distribution | Mann-Whitney test | 0.42 | Power | 0.209 |
|  |  |  |  | Effect size | -0.390 |
|  |  |  |  | 95% C.I. of diff | -0.878 to 0.294 |

| Sag amplitude at -100  **Panel H** | Data structure | test | P value |  | |
| --- | --- | --- | --- | --- | --- |
| **EGFP^+^ vs EGFP^-^ P4/5** | Normal distribution | Two-sample 2-tailed t - test | 0.55 | Power | 0.09094 |
|  |  |  |  | Effect size | -0.999 to 0.575 |
|  |  |  |  | 95% C.I. of diff | -0.222 |
| **EGFP^+^ vs EGFP^-^ P10/11** | Normal distribution | Two-sample 2-tailed t - test | 0.06 |  |  |
|  |  |  |  | Effect size | -0.787 |
|  |  |  |  | 95% C.I. of diff | -1.37 to -0.031 |
| **EGFP^+^ vs EGFP^-^ P14/15** | Non Normal distribution | Mann-Whitney test | 0.025 | Power | 0.38 |
|  |  |  |  | Effect size | -0.567 |
|  |  |  |  | 95% C.I. of diff | -1.05 to 0.216 |
