# Supplementary material for: Basal Forebrain Cholinergic Neurons Have Specific Characteristics during the Perinatal Period
Source: eNeuro. 2024 May 24;11(5):ENEURO.0538-23.2024. doi: 10.1523/ENEURO.0538-23.2024 (PMC11137802; doi:10.1523/ENEURO.0538-23.2024)
Supplement: Table 4-1 — Statistical analysis related to Figure 4 and Extended Data Figure 4-2. Summary of statistical tests for Figure 4 F-H and Extended Data Figure 4-2 F-H. LCL-Lower Confidence Interval, UCL- Upper Confidence Interval Download Table 4-1, DOCX file. [file eneuro-11-ENEURO.0538-23.2024-s007.docx]

**Extended Data Table 4-1**

Statistical analysis related to **Figure 4**

| Groups | n (cells) | N (mice) | Delay 1st spike (ms)  **Panel F** | Max Frequency (Hz)  **Panel G** | AHP (mV)  **Panel H** |
| --- | --- | --- | --- | --- | --- |
| **EGFP^+^ P2-5** | 23 | 11 | 245.7 ± 29.5 | 16.39 ± 1.06 | -14.33± 0.98 |
| **EGFP^+^1 P12-P15** | 18 | 9 | 215.2 ± 16.5 | 14.89 ±1.95 | -12.98± 1.68 |
| **EGFP^+^2 P12-P15** | 12 | 6 | 696.7 ± 49.5 | 7.17 ± 0.52 | -20.33±1.26 |

| Groups  **Panel F** | Data structure | Univariate ANOVA  between groups | Fisher’s LSD Post-hoc test  between the groups | LCL-UCL |
| --- | --- | --- | --- | --- |
| delay1stspike **EGFP^+^**1P12-15 vs delay1stspike **EGFP^+^**P2-5 | Normal distribution | 6*e^-14^ | 0.45 | -112.8 51.7 |
| delay1stspike **EGFP^+^**2 P12-15 vs delay1stspike **EGFP^+^** P2-5 | Normal distribution |  | 4*e^-13^ | 357.8 544.0 |
| delay1stspike **EGFP^+^**2 P12-15 vs delay1stspike **EGFP^+^**1 P12-15 | Normal distribution |  | 2*e^-13^ | 384.07 578.9 |

| Groups  **Panel G** | Data structure | Univariate ANOVA  between groups | Fisher’s LSD Post-hoc test  between the groups | LCL-UCL |
| --- | --- | --- | --- | --- |
| Max Frequency **EGFP^+^**1P12-15 vs Max Frequency **EGFP^+^**P2-5 | Normal distribution | 2*e^-4^ | 0.43 | -5.27 2.26 |
| Max Frequency **EGFP^+^**2 P12-15 vs Max Frequency **EGFP^+^** P2-5 | Normal distribution |  | 7*e^-5^ | -13.48 -4.96 |
| Max Frequency **EGFP^+^**2 P12-15 vs Max Frequency **EGFP^+^**1 P12-15 | Normal distribution |  | 1*e^-3^ | -12.18 -3.26 |

| Groups  **Panel H** | Data structure | Kruskall-Wallis test | Dunn’s adj. p value |
| --- | --- | --- | --- |
| AHP **EGFP^+^**1P12-15 vs  AHP **EGFP^+^**P2-5 | Normal distribution | 0.0027 | > 0,9999 |
| AHP **EGFP^+^**2 P12-15 vs  AHP **EGFP^+^** P2-5 | Normal distribution |  | 0.015 |
| AHP **EGFP^+^**2 P12-15 vs  AHP **EGFP^+^**1 P12-15 | Normal distribution |  | 0.0028 |

Statistical analysis related **Extended data Figure 4-2**

| Groups | n (cells) | N (mice) | Delay 1st spike (ms)  **Panel F** | Max Frequency (Hz)  **Panel G** | AHP (mV)  **Panel H** |
| --- | --- | --- | --- | --- | --- |
| **EGFP^+^ P2-5** | 23 | 11 | 245.7 ± 29.5 | 16.39 ± 1.06 | -14.33± 0.98 |
| **EGFP^+^1 P12-P15** | 18 | 9 | 215.2 ± 16.5 | 14.89 ±1.95 | -12.98± 1.68 |
| **EGFP^+^2 P12-P15** | 12 | 6 | 696.7 ± 49.5 | 7.17 ± 0.52 | -20.33± 1.26 |
| **EGFP^-^ P2-5** | 14 | 3 | 214.3 ± 29.6 | 14.93 ± 2.893 | -17.81 ± 1.191 |
| **EGFP^-^ 1 P12-P15** | 14 | 6 | 270.4 ± 22.8 | 10.21 ± 1.52 | -13.46 ± 1.24 |
| **EGFP^-^ 2 P12-P15** | 4 | 3 | 639.5 ± 19.2 | 5.75 ± 0.63 | -21.50 ± 0.96 |

| Groups  **Panel F** | Data structure | Univariate ANOVA  between groups | Fisher’s LSD Post-hoc test  between the groups | LCL-UCL |
| --- | --- | --- | --- | --- |
| delay1stspike **EGFP^+^**P2-5 vs delay1stspike **EGFP^-^** P2-5 | Normal distribution | 2*e^-19^ | 0.44 | -111.2, 48.38 |
| delay1stspike **EGFP^+^ 1** P12-15 vs delay1stspike **EGFP^-^ 1** P12-15 | Normal distribution |  | 0.2 | -28.62, 139.1 |
| delay1stspike **EGFP^+^ 2** P12-15 vs delay1stspike **EGFP^-^ 2** P12-15 | Normal distribution |  | 0.4 | -193.1, 78.74 |
| delay1stspike **EGFP^+^**1P12-15 vs delay1stspike **EGFP^+^**P2-5 | Normal distribution |  | 0.41 | -104.7, 43.5 |
| delay1stspike **EGFP^+^**2 P12-15 vs delay1stspike **EGFP^+^** P2-5 | Normal distribution |  | 5*e^-17^ | 367.1, 534.7 |
| delay1stspike **EGFP^+^**2 P12-15 vs delay1stspike **EGFP^+^**1 P12-15 | Normal distribution |  | 2*e^-17^ | 393.8, 569.2 |
| delay1stspike **EGFP^-^**1P12-15 vs delay1stspike **EGFP^-^** P2-5 | Normal distribution |  | 0.2 | -32.87, 145.1 |
| delay1stspike **EGFP^-^**2 P12-15 vs delay1stspike **EGFP^-^** P2-5 | Normal distribution |  | 1*e^-8^ | 291.7, 558.6 |
| delay1stspike **EGFP^-^**2 P12-15 vs delay1stspike **EGFP^-^**1 P12-15 | Normal distribution |  | 5*e^-7^ | 235.6, 502.5 |

| Groups  **Panel G** | Data structure | Univariate ANOVA  between groups | Fisher’s LSD Post-hoc test  between the groups | LCL-UCL |
| --- | --- | --- | --- | --- |
| Max Frequency **EGFP^+^**P2-5 vs  Max Frequency **EGFP^-^** P2-5 | Normal distribution | 8*e^-4^ | 0.53 | -6.09, 3.17 |
| Max Frequency **EGFP^+^ 1** P12-15 vs Max Frequency **EGFP^-^ 1** P12-15 | Normal distribution |  | 0.059 | -9.54, 0.19 |
| Max Frequency **EGFP^+^ 2** P12-15 vs Max Frequency **EGFP^-^ 2** P12-15 | Normal distribution |  | 0.72 | -9.30, 6.47 |
| Max Frequency **EGFP^+^**1P12-15 vs Max Frequency **EGFP^+^**P2-5 | Normal distribution |  | 0.0096 | -10.8, -1.54 |
| Max Frequency **EGFP^+^**2 P12-15 vs Max Frequency **EGFP^+^** P2-5 | Normal distribution |  | 3*e^-4^ | -14.09, -4.36 |
| Max Frequency **EGFP^+^**2 P12-15 vs Max Frequency **EGFP^+^**1 P12-15 | Normal distribution |  | 0.003 | -12.8, -2.63 |
| Max Frequency **EGFP^-^**1P12-15 vs Max Frequency **EGFP^-^** P2-5 | Normal distribution |  | 0.073 | -9.88, 0.45 |
| Max Frequency **EGFP^-^**2 P12-15 vs Max Frequency **EGFP^-^** P2-5 | Normal distribution |  | 0.005 | -13.14, -2.38 |
| Max Frequency e **EGFP^-^**2 P12-15 vs Max Frequency **EGFP^-^**1 P12-15 | Normal distribution |  | 0.25 | -12.21, 3.28 |

| Groups  **Panel H** | Data structure | Kruskall-Wallis test | Dunn’s adj. p value |
| --- | --- | --- | --- |
| AHP **EGFP^+^**P2-5 vs  AHP **EGFP^-^** P2-5 | Normal distribution | 0.0002 | 0.6158 |
| AHP **EGFP^+^ 1** P12-15 vs  AHP **EGFP^-^ 1** P12-15 | Normal distribution |  | > 0,9999 |
| AHP **EGFP^+^ 2** P12-15 vs  AHP **EGFP^-^ 2** P12-15 | Normal distribution |  | > 0,9999 |
| AHP **EGFP^+^**1P12-15 vs  AHP **EGFP^+^**P2-5 | Normal distribution |  | > 0,9999 |
| AHP **EGFP^+^**2 P12-15 vs  AHP **EGFP^+^** P2-5 | Normal distribution |  | 0.0454 |
| AHP **EGFP^+^**2 P12-15 vs  AHP **EGFP^+^**1 P12-15 | Normal distribution |  | 0.0104 |
| AHP **EGFP^-^**1P12-15 vs  AHP **EGFP^-^** P2-5 | Normal distribution |  | 0.418 |
| AHP **EGFP^-^**2 P12-15 vs  AHP **EGFP^-^** P2-5 | Normal distribution |  | > 0,9999 |
| AHP **EGFP^-^**2 P12-15 vs  AHP **EGFP^-^**1 P12-15 | Normal distribution |  | 0.0356 |
