# Supplementary material for: Basal Forebrain Cholinergic Neurons Have Specific Characteristics during the Perinatal Period
Source: eNeuro. 2024 May 24;11(5):ENEURO.0538-23.2024. doi: 10.1523/ENEURO.0538-23.2024 (PMC11137802; doi:10.1523/ENEURO.0538-23.2024)
Supplement: Table 5-1 — Statistical analysis related to Figure 5. Summary of statistical tests for Figure 5C. 95% C.I. of diff - confidence interval for effect size, LCL-Lower Confidence Interval, UCL- Upper Confidence Interval. Download Table 5-1, DOCX file. [file eneuro-11-ENEURO.0538-23.2024-s008.docx]

**Extended Data Table 5-1**

Statistical analysis related to **Figure 5, panel C**

| Groups | n (cells) | N (mice) | Spike frequency (normalized to control)  Mean ± SEM | Data structure | Paired two-tail t-test | Powers | |
| --- | --- | --- | --- | --- | --- | --- | --- |
| **P2/3** | 9 | 3 | 2.65±0.38 | Normal distribution | 0.0026 | Effect size | 2.16 |
|  |  |  |  |  |  | 95% C.I. of diff | 1.124 to 2.60 |
| **P4/5** | 12 | 5 | 2.52±0.34 | Normal distribution | 1*e^-3^ | Effect size | 1.89 |
|  |  |  |  |  |  | 95% C.I. of diff | 0.99 to 2.324 |
| **P6/7** | 16 | 4 | 1.77±0.14 | Normal distribution | 5*e^-5^ | Effect size | 2.05 |
|  |  |  |  |  |  | 95% C.I. of diff | 0.547 to 1.064 |
| **P8/9** | 12 | 4 | 0.82±0.05 | Normal distribution | 0.0084 | Effect size | 1.37 |
|  |  |  |  |  |  | 95% C.I. of diff | -0.28 to -0.077 |
| **P10/11** | 12 | 3 | 0.67±0.25 | Normal distribution | 0.21 | Powers | 0.23036 |
|  |  |  |  |  |  | Effect size | 0.57 |
|  |  |  |  |  |  | 95% C.I. of diff | -0.726 to 0.236 |
| **P14/15** | 7 | 4 | 0.50±0.10 | Normal distribution | 0.00265 | Effect size | 2.84 |
|  |  |  |  |  |  | 95% C.I. of diff | -0.67 to -0.313 |

| Spike frequency (normalized to control)  Groups  **Panel C** | Data structure | Univariate ANOVA  between groups | Fisher’s LSD Post-hoc test  between the groups | Powers | LCL-UCL |
| --- | --- | --- | --- | --- | --- |
| **P2/3 vs P4/5** | Normal distribution | 1*E^-8^ | 0.73 | 1 | -0.83, 0.58 |
| **P4/5 vs P6/7** | Normal distribution |  | 0.016 |  | -1.36, -0.14 |
| **P6/7 vs P8/9** | Normal distribution |  | 0.003 |  | -1.55, -0.33 |
| **P8/9 vs P10/11** | Normal distribution |  | 0.63 |  | -0.81, 0.49 |
| **P10/11 vs P14/15** | Normal distribution |  | 0.67 |  | -0.921, 0.59 |
| **P4/5 vs P10/11** | Normal distribution |  | 4*e^-7^ |  | -2.51, -1.20 |
| **P4/5 vs P14/15** | Normal distribution |  | 2*e^-6^ |  | -2.78, -1.25 |
