# Supplementary material for: Basal Forebrain Cholinergic Neurons Have Specific Characteristics during the Perinatal Period
Source: eNeuro. 2024 May 24;11(5):ENEURO.0538-23.2024. doi: 10.1523/ENEURO.0538-23.2024 (PMC11137802; doi:10.1523/ENEURO.0538-23.2024)
Supplement: Table 6-1 — Statistical analysis related to Figure 6. Summary of statistical tests for Figure 6 B,C. 95% C.I. of diff - confidence interval for effect size. Download Table 6-1, DOCX file. [file eneuro-11-ENEURO.0538-23.2024-s009.docx]

**Extended Data Table 6-1**

Statistical analysis related to **Figure 6**

| Groups | n (cells) | N (mice) | GABAergic PSCs Amplitude (pA)  Mean ± SEM  **Panel B** | GABAergic PSCs Frequency (Hz)  Mean ± SEM  **Panel C** |
| --- | --- | --- | --- | --- |
| **P0** | 7 | 5 | 59.96±8.71 | 1.016±0.33 |
| **P4/5** | 9 | 7 | 41.94±4.74 | 2.50±0.53 |
| **P10/11** | 8 | 4 | 54.54±7.39 | 10.59±3.01 |
| **P14/15** | 9 | 6 | 42.88±2.82 | 13.11±2.87 |

| GABAergic PSCs Amplitude  Groups  **Panel B** | Data structure | test | P value |  | |
| --- | --- | --- | --- | --- | --- |
| **P0/1 vs P4/5** | Normal distribution | Two-sample 2-tailed t - test | 0.07 | Power | 0.43 |
|  |  |  |  | Effect size | 1.04 |
|  |  |  |  | 95% C.I. of diff | -2.08, 0.218 |
| **P4/5 vs P14/15** | Normal distribution | Two-sample 2-tailed t - test | 0.86 | Power | 0.05 |
|  |  |  |  | Effect size | 0.08 |
|  |  |  |  | 95% C.I. of diff | -0.867, 1.07 |

| GABAergic PSCs Frequency  Groups  **Panel C** | Data structure | test | Adjusted P value* |  | |
| --- | --- | --- | --- | --- | --- |
| **P0/1 vs P4/5** | Normal distribution | Two-sample 2-tailed t - test | 0.04 |  |  |
|  |  |  |  | Effect size | 1.20 |
|  |  |  |  | 95% C.I. of diff | 0.145, 1.99 |
| **P4/5 vs P14/15** | Normal distribution | Two-sample 2-tailed t - test | 0.004 |  |  |
|  |  |  |  | Effect size | 1.82 |
|  |  |  |  | 95% C.I. of diff | 1.06, 2.3 |

*- Holm-Bonferroni Sequential Correction: An EXCEL Calculator" © Justin Gaetano, 2013
