# Supplementary material for: Basal Forebrain Cholinergic Neurons Have Specific Characteristics during the Perinatal Period
Source: eNeuro. 2024 May 24;11(5):ENEURO.0538-23.2024. doi: 10.1523/ENEURO.0538-23.2024 (PMC11137802; doi:10.1523/ENEURO.0538-23.2024)
Supplement: Table 7-1 — Statistical analysis related to Figure 7. Summary of statistical tests for Figure 7 B,C. 95% C.I. of diff - confidence interval for effect size. Download Table 7-1, DOCX file. [file eneuro-11-ENEURO.0538-23.2024-s010.docx]

**Extended Data Table 7-1**

Statistical analysis related to **Figure 7**

| Groups | n (cells) | N (mice) | **Glutamat**ergic PSCs Amplitude (pA)  Mean ± SEM  **Panel B** | **Glutamat**ergic PSCs Frequency (Hz)  Mean ± SEM  **Panel C** |
| --- | --- | --- | --- | --- |
| **P0** | 9 | 6 | 5.44±1.48 | 1.21± 0.30 |
| **P4/5** | 12 | 6 | 7.06±1.78 | 4.20±0.89 |
| **P10/11** | 12 | 7 | 7.82±3.76 | 6.18±2.63 |
| **P14/15** | 10 | 4 | 8.75±2.69 | 9.21±1.15 |

| Glutamatergic PSCs Amplitude  Groups  **Panel B** | Data structure | test | Adjusted P value |  | |
| --- | --- | --- | --- | --- | --- |
| **P0/1 vs P4/5** | Normal distribution | Two-sample 2-tailed t - test | 0.078 | Power | 0.56 |
|  |  |  |  | Effect size | 0.937 |
|  |  |  |  | 95% C.I. of diff | -0.0713, 1.71 |
| **P4/5 vs P14/15** | Normal distribution | Two-sample 2-tailed t - test | 0.09 | Power | 0.39 |
|  |  |  |  | Effect size | 0.757 |
|  |  |  |  | 95% C.I. of diff | -0.204, 1.68 |

| Glutamatergic PSCs Frequency  Groups  **Panel C** | Data structure | test | Adjusted P value* |  | |
| --- | --- | --- | --- | --- | --- |
| **P0/1 vs P4/5** | Normal distribution | Two-sample 2-tailed t - test | 0.01 |  |  |
|  |  |  |  | Effect size | 1.18 |
|  |  |  |  | 95% C.I. of diff | 0.402, 1.72 |
| **P4/5 vs P14/15** | Normal distribution | Two-sample 2-tailed t - test | 0.004 |  |  |
|  |  |  |  | Effect size | 1.23 |
|  |  |  |  | 95% C.I. of diff | 0.419, 1.79 |

*- Holm-Bonferroni Sequential Correction: An EXCEL Calculator" © Justin Gaetano, 2013
